# Optimal selection of ovarian stimulation protocol for infertile women with diminished ovarian reserve based on Bologna and POSEIDON criteria: a network meta-analysis

**DOI:** 10.1186/s12958-026-01573-6

**Published:** 2026-06-05

**Authors:** Ting Zhu, Jie Zhang, Xiaoyu Zhang, Yutian Zhou, Chaoyi Wang, Yi Qian, Jinyong Liu, Dan Zhou, Juan Jiang, Yan Meng

**Affiliations:** 1 State Key Laboratory of Reproductive Medicine and Offspring Health, Clinical Center of Reproductive Medicine, The First Affiliated Hospital with Nanjing Medical University, Nanjing, 210029 China; 2 The Affiliated Suqian First People’s Hospital of Nanjing Medical University, Suqian, 223800 China; 3 Liyang Branch of Jiangsu Provincial Hospital of Chinese Medicine, Liyang Hospital of Chinese Medicine, Changzhou, 213000 China; 4 JingJiang People’s Hospital, Taizhou, 214500 China

**Keywords:** Diminished ovarian reserve, IVF, Ovarian stimulation protocol, POSEIDON criteria, Bologna criteria

## Abstract

**Background:**

Patients with diminished ovarian reserve (DOR) present a significant challenge in assisted reproductive technology due to their poor ovarian response and lower pregnancy rates. Although multiple ovarian stimulation protocols are available, the optimal regimen for DOR patients remains uncertain, largely due to population heterogeneity and inconsistent outcome reporting.

**Purpose:**

To compare the efficacy of five commonly used controlled ovarian stimulation protocols, including GnRH agonist, GnRH antagonist, mild stimulation, progestin-primed ovarian stimulation (PPOS), and double ovarian stimulation (DOS), in patients with DOR, using a network meta-analysis stratified by Bologna and POSEIDON criteria.

**Methods:**

This was a systematic review and network meta-analysis including randomized controlled trials and retrospective cohort studies identified through searches of PubMed, Cochrane Library, Web of Science, and Embase from inception to November 2024. The analysis involved studies comparing various controlled ovarian hyperstimulation (COH) regimens in DOR patients. The included studies enrolled women diagnosed with DOR based on Bologna or POSEIDON criteria. The primary outcome was live birth rate at the first embryo transfer (ET) cycle, while secondary outcomes included the number of oocytes retrieved, clinical pregnancy rate at first ET, cumulative live birth rate, and cycle cancellation rate. Network meta-analyses were performed using STATA V.14.0 and R V.4.3.2 to compare outcomes across different stimulation protocols within stratified patient groups.

**Results:**

In the Bologna group, the double ovarian stimulation (DOS) protocol demonstrated significantly higher effectiveness in terms of live birth rates. Additionally, it yielded higher oocyte counts and clinical pregnancy rates, while also resulting in lower cycle cancellation rates. However, under the POSEIDON criteria, the results varied. For patients categorized in POSEIDON group 3, the GnRH agonist protocol achieved the highest live birth rate, significantly outperforming mild stimulation (OR 7.69, 95% CI: 3.57–16.67) and GnRH antagonist (OR 1.97, 95% CI: 1.36–2.85). The GnRH agonist protocol also led in oocyte retrieval, cumulative live birth rate, and clinical pregnancy rate, and showed a lower cycle cancellation risk. In contrast, for POSEIDON group 4 patients, no significant differences in live birth rates, clinical pregnancy rates or cycle cancellation risk were observed among protocols. While DOS resulted in higher oocyte retrieval than other protocols, GnRH agonist showed a higher cumulative live birth rate than GnRH antagonist (OR 1.25, 95% CI: 1.04–1.50).

**Conclusions:**

This analysis highlights the importance of individualized stimulation protocols based on patient stratification. For DOR patients, especially those classified using POSEIDON criteria, tailoring ovarian stimulation strategies could enhance clinical outcomes. Future prospective trials are warranted to refine protocol recommendations for specific DOR subgroups.

**Supplementary Information:**

The online version contains supplementary material available at 10.1186/s12958-026-01573-6.

## Introduction

Diminished ovarian reserve (DOR) poses a significant challenge in assisted reproductive technology (ART), often resulting in reduced ovarian response, poor IVF/ICSI outcomes, and increased emotional and financial burdens for patients. Characterized by elevated follicle-stimulating hormone (FSH) levels, reduced anti-Mullerian hormone (AMH) levels, and limited oocyte retrieval, DOR frequently necessitates multiple costly IVF cycles and increases the likelihood of treatment failures. These challenges underscore the urgent need for optimized controlled ovarian hyperstimulation (COH) strategies tailored to this population.

Over recent decades, a variety of ovarian stimulation protocols have been developed to improve pregnancy outcomes in DOR patients, including GnRH agonist and antagonist protocols, mild stimulation protocols [1], progestin-primed ovarian stimulation (PPOS) [2], and double ovarian stimulation (DOS) protocols [3]. However, the lack of consensus on the optimal COH regimen remains a pressing issue, primarily due to the heterogeneity in patient profiles. To address this limitation, the Bologna criteria [4] were developed to standardize the definition of DOR, focusing on markers such as AMH < 1.1 ng/mL or AFC < 5–7. While useful, these criteria encompass a broad range of patient phenotypes. The POSEIDON classification [5], was subsequently proposed to offer a more refined stratification by incorporating the ovarian sensitivity as well as oocyte quality. It categorizes patients into four groups; among them, Groups 3 and 4 include those with poor ovarian reserve (AMH < 1.2 ng/mL or AFC < 5), further stratified by age ≤ 35 years (Group 3) or > 35 years (Group 4). Nevertheless, its clinical utility remains to be fully validated.

Traditional pairwise meta-analyses [6–10], while useful, are limited to direct comparisons between two protocols, leaving many treatment options unexplored. Network meta-analysis offers a solution by enabling simultaneous comparison of multiple treatments within a unified framework, combining direct and indirect evidence to improve the precision of effect estimates. This approach not only facilitates the ranking of COH protocols but also highlights knowledge gaps, guiding future research priorities.

In this study, we performed a systematic review and network meta-analysis to compare the efficacy of commonly used ovarian stimulation protocols in patients with DOR. Studies were categorized using the Bologna criteria and further stratified by POSEIDON classification based on patient age. We aimed to provide clinicians with a ranked list of protocols, thereby facilitating more informed and personalized treatment decisions.

## Methods

### Study design and registration

This systematic review and network meta-analysis was conducted in compliance with the PRISMA guidelines [11], and the protocol was registered in the PROSPERO database (ID: CRD42023396818).

## Search strategy and study selection

A comprehensive literature search was performed in PubMed, Cochrane Library, Web of Science, and Embase databases from their inception to November 2024. The search strategy employed Medical Subject Headings (MeSH) terms (https://www.nlm.nih.gov/mesh/MBrowser.html) and free-text keywords including: (i) “diminished ovarian reserve” or “DOR” or “poor ovarian response” or “POR” or “POSEIDON criteria” or “Bologna criteria” or “ovarian reserve” or “anti-Müllerian hormone” or “AMH” or “antral follicle count” or “AFC”, (ii) “ovulation induction” OR “ovarian stimulation” OR “controlled ovarian stimulation” OR “COS” OR “Controlled ovarian hyperstimulation” OR “COH”, (iii) “in vitro fertilization outcome” OR “IVF outcome” OR “intracytoplasmic sperm injection outcome” OR “ICSI outcome” OR “IVF/ICSI outcome” OR “ART outcome”. These subsets were combined using “AND” to generate a subset of citations relevant to the research subject.

Eligible studies were independently screened and evaluated by two investigators (Z.T. and X.Y.Z.). Studies were included if they were RCTs or non-RCTs involving patients diagnosed with DOR, defined by basal antral follicle count (AFC) and AMH levels.

Included studies compared different ovarian stimulation protocols and reported prespecified clinical outcomes of interest. Exclusion criteria were as follows: comparisons limited to different doses or durations of the same protocol, studies reporting cumulative outcomes from multiple IVF/ICSI cycles, and studies lacking full-text availability. Reviews, conference abstracts, letters, and case reports were also excluded.

Studies were classified according to ovarian reserve and age-based criteria.

Specifically, studies that included patients with AFC < 5–7 or AMH < 1.1–1.2 ng/mL, regardless of age, were categorized under the Bologna group. Studies including women aged > 35 years were assigned to POSEIDON group 4, while those with participants aged ≤ 35 years were classified as POSEIDON group 3. In studies lacking explicit age thresholds, the 90th percentile of patient age was estimated using the reported mean (µ) and standard deviation (σ) to assign the appropriate POSEIDON subgroup.

The primary outcome was the live birth rate (LBR) in first embryo transfer, defined as a baby born alive. Secondary outcomes included the number of oocytes retrieved, clinical pregnancy rate in first embryo transfer (presence of gestational sac or fetal heartbeat via ultrasonography), cumulative live birth rate, and cycle cancellation rate (cycle canceled before oocyte retrieval).

### Data extraction and quality assessment

Two investigators (Z.T. and X.Y.Z.) independently extracted data using a standardized form. Extracted information included authors, publication year, country, studied groups, trial design, sample size, inclusion/exclusion criteria, age, infertility duration, AMH, AFC, specifics of the COH protocols, adjuvant treatments, and IVF/ICSI outcomes. Continuous variables reported as medians and quartiles (or ranges) were converted to means and standard deviations (SDs) using established methods by Luo et al. [12] Ambiguous or incomplete data were clarified by contacting corresponding authors. Discrepancies were resolved by consensus or discussion with a third reviewer (M.Y.).

Study quality was independently evaluated by Z.T. and X.Y.Z. using the revised Cochrane risk of bias tool for randomized trials (RoB2) for assessing RCTs, and the Newcastle-Ottawa Scale (NOS) tool for non-RCTs, with a score ≥ 6 stars indicating high-quality studies.

### Data synthesis and statistical analysis

Statistical analyses were performed using odds ratios (ORs) for dichotomous outcomes and mean differences (MDs) for continuous outcomes, with corresponding 95% confidence intervals (CIs). Dichotomous outcome data were pooled to determine the OR with corresponding 95% CIs. Continuous outcome data were pooled using the inverse variance model, and MDs with 95% CIs were calculated to determine the effect size. A network meta-analysis was conducted using the “mvmeta” package in STATA (version 14.0) with a random-effects multiple regression model to compare multiple ovarian stimulation protocols simultaneously. A network plot was constructed to visualize the geometry of treatment comparisons.

An inconsistency plot was used to assess consistency between direct and indirect evidence. Treatment efficacy was ranked using the surface under the cumulative ranking curve (SUCRA), where higher SUCRA values indicated greater treatment effectiveness [13]. The efficacy of each intervention was expressed as a percentage relative to an imaginary best-performing intervention. Publication and small-study biases were assessed using comparison-adjusted funnel plots, with visual inspection for asymmetry to detect potential biases [14].

## Results

### Study selection and characteristics

**Fig. 1:**
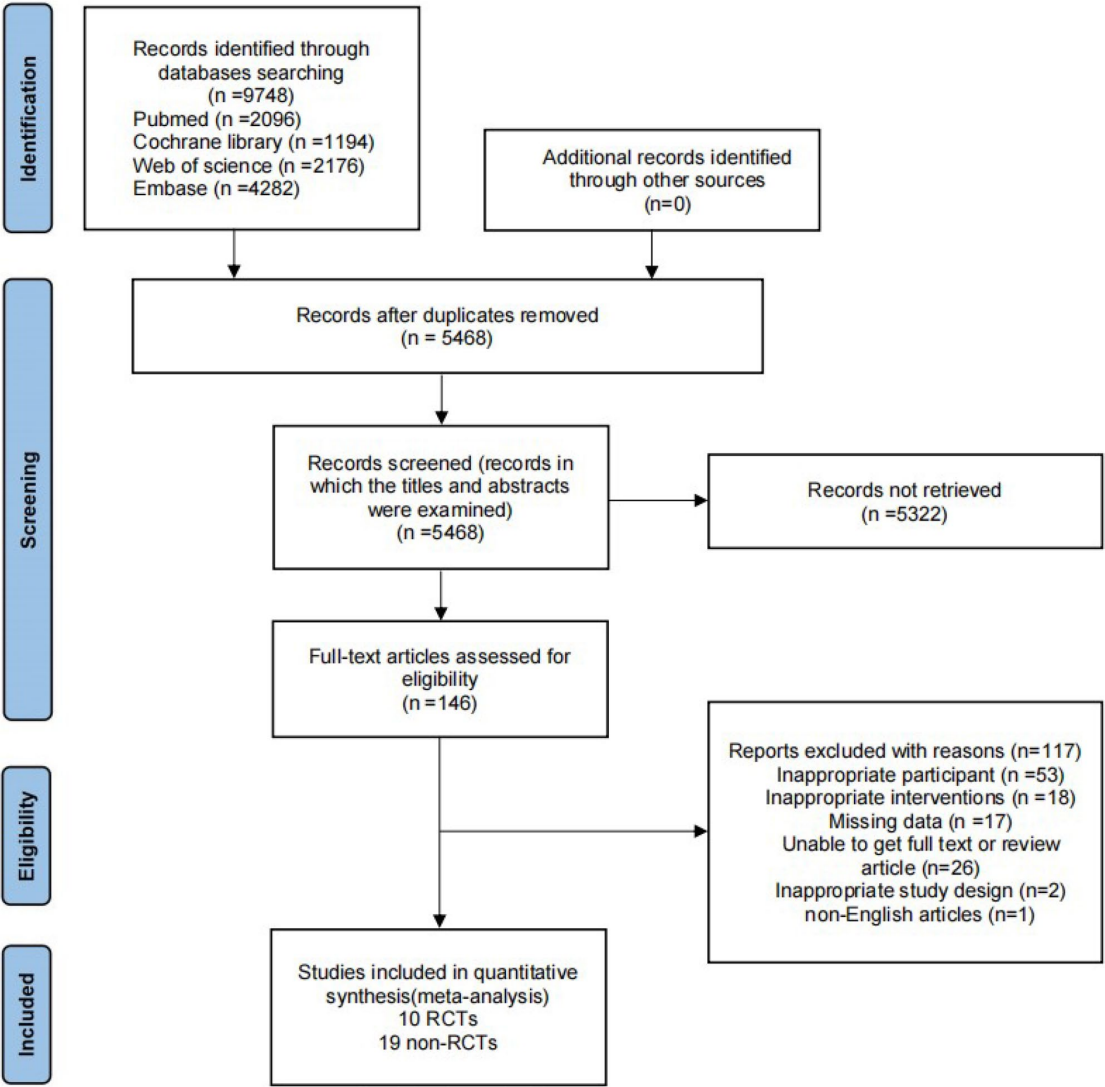
PRISMA flow chart of the study retrieval and selection

The study selection process was showed in Fig. 1. A total of 9,748 publications were identified in accordance with the unrestricted literature search yielded, with 3,980 excluded for duplications. After we screened the titles and abstracts of these manuscripts, 146 potentially qualified articles were considered to check eligibility. These 146 studies were further evaluated by reading the full text. At last, 117 studies were excluded. Finally, 10 RCTs [10, 15–23] and 19 non-RCT studies [24–42] were included in the network meta-analysis. The characteristics of all the studies included are presented in Table S2.

A total of 29 trials were included, comparing five ovarian stimulation protocols with at least two arms each, including GnRH agonist, GnRH antagonist, mild stimulation, PPOS, and DOS protocols. The overall risk-of-bias assessment for the 10 RCT studies in the network meta-analysis indicated that 1 had high risk and 4 had some concerns of deviations from intended interventions, 4 had concerns about the randomization process; 4 were at low risk, 5 were at some concerns and 1had high risk with complete outcome data, as detailed in Figure S1. Non-RCTs evaluated with the NOS tool exhibited inevitable selection bias, and 19 rated as high-quality evidence (Table S3). In subgroup analysis, POSEIDON group 3 comprised 6 non-RCTs, while POSEIDON group 4 included 4 RCTs and 13 non-RCTs. Figure S2 presented the network plots for the primary and secondary outcomes.

### Bologna group

**Fig. 2:**
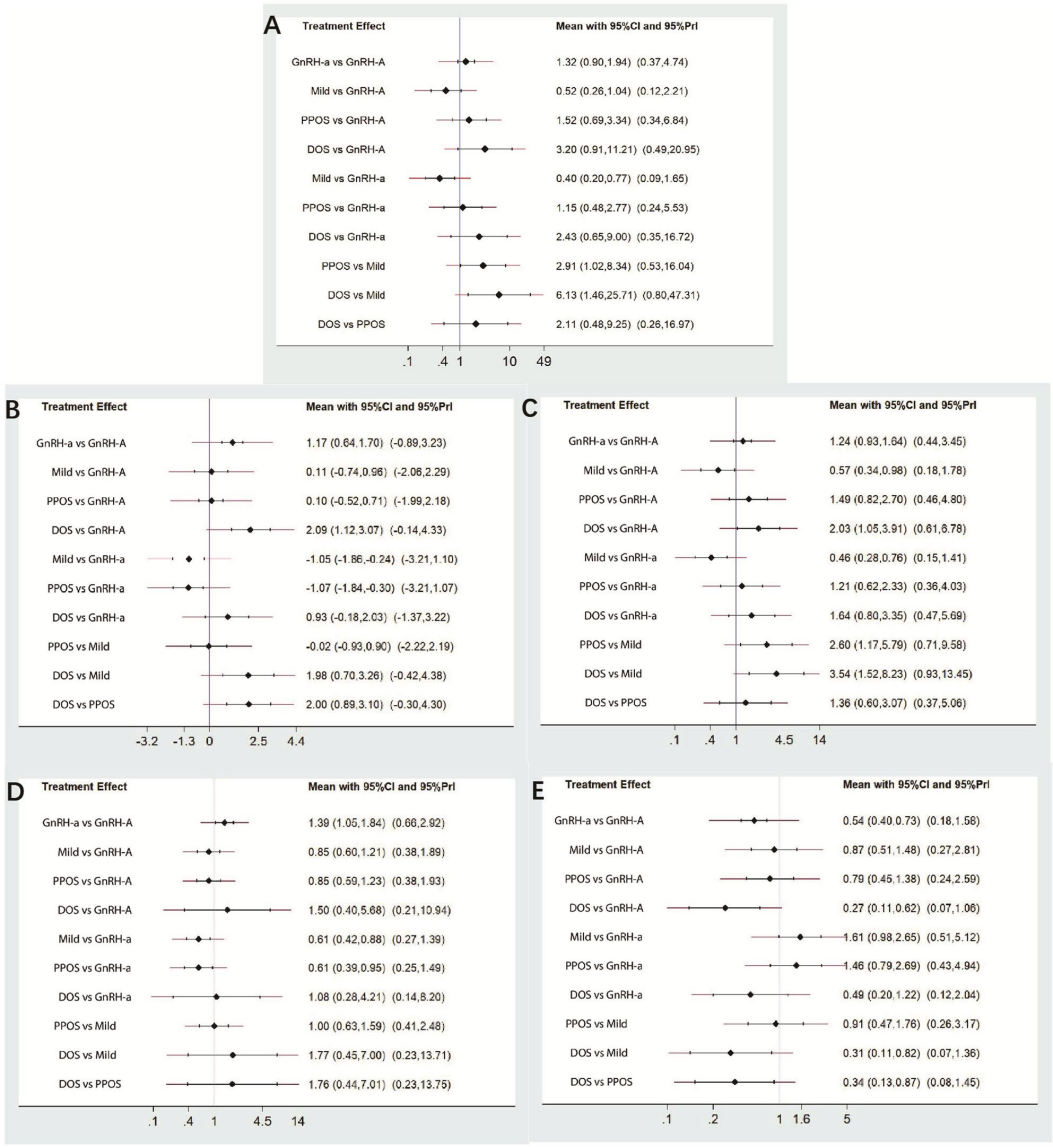
Forest plots of network meta-analyses of the Bologna group. **A **Live birth rate; **(B)** The number of oocytes retrieved; **(C)** Clinical pregnancy rate; **(D)** Cumulative Live birth rate; **(E)** Cycle cancellation rate

Regarding the live birth rate, mild stimulation was significantly less effective compared to DOS (OR 0.16, 95% CI: 0.04–0.68), PPOS (OR 0.34, 95% CI: 0.12–0.98), and GnRH agonist (OR 0.40, 95% CI: 0.20–0.77) (Fig. 2A). However, no statistically significant differences were observed between the other protocols. The SUCRA values for live birth rate were as follows: 92.4% for DOS, 65.3% for PPOS, 59.9% for GnRH agonist, 30.8% for GnRH antagonist, and 1.7% for mild stimulation (Fig. S3). In terms of oocyte retrieval, DOS and GnRH agonist protocols yielded significantly higher counts compared to other stimulation protocols (Fig. 2B). The SUCRA values for oocyte retrieval were 98.7% for DOS, 76% for GnRH agonist, 28.1% for mild stimulation, 27.9% for PPOS, and 19.2% for GnRH antagonist (Fig. S3). With regard to clinical pregnancy rate, mild stimulation was significantly less effective than DOS (OR 0.28, 95% CI: 0.12–0.66), PPOS (OR 0.46, 95% CI: 0.28–0.76), and GnRH agonist (OR 0.57, 95% CI: 0.34–0.98) (Fig. 2C). The SUCRA values for clinical pregnancy rate were 92% for DOS, 70.7% for PPOS, 57.7% for GnRH agonist, 29% for GnRH antagonist, and 0.7% for mild stimulation (Fig. S3).

For cumulative live birth rate, GnRH agonist ranked the highest, showing significant advantages over GnRH antagonist (OR 1.39, 95% CI: 1.05–1.84), PPOS (OR 1.64, 95% CI: 1.05–2.56), and mild stimulation (OR 1.64, 95% CI: 1.14–2.38) (Fig. 2D).

The SUCRA values for cumulative live birth rate were 85.6% for GnRH agonist, 71.4% for DOS, 47.6% for GnRH antagonist, 23.3% for PPOS, and 22.1% for mild stimulation (Fig. S3). In terms of the risk of cycle cancellation, DOS was associated with a significantly lower risk compared to GnRH antagonist (OR 0.27, 95% CI: 0.11–0.62), mild stimulation (OR 0.31, 95% CI: 0.11–0.82), and PPOS (OR 0.34, 95% CI: 0.13–0.87) (Fig. 2E). Additionally, GnRH agonist demonstrated a lower risk of cycle cancellation than GnRH antagonist (OR 0.54, 95% CI: 0.40–0.73). The SUCRA values for the risk of cycle cancellation were 87.8% for GnRH antagonist, 72.1% for mild stimulation, 61% for PPOS, 27.2% for GnRH agonist, and 1.9% for DOS (Fig. S3).

### POSEIDON group 3

**Fig. 3:**
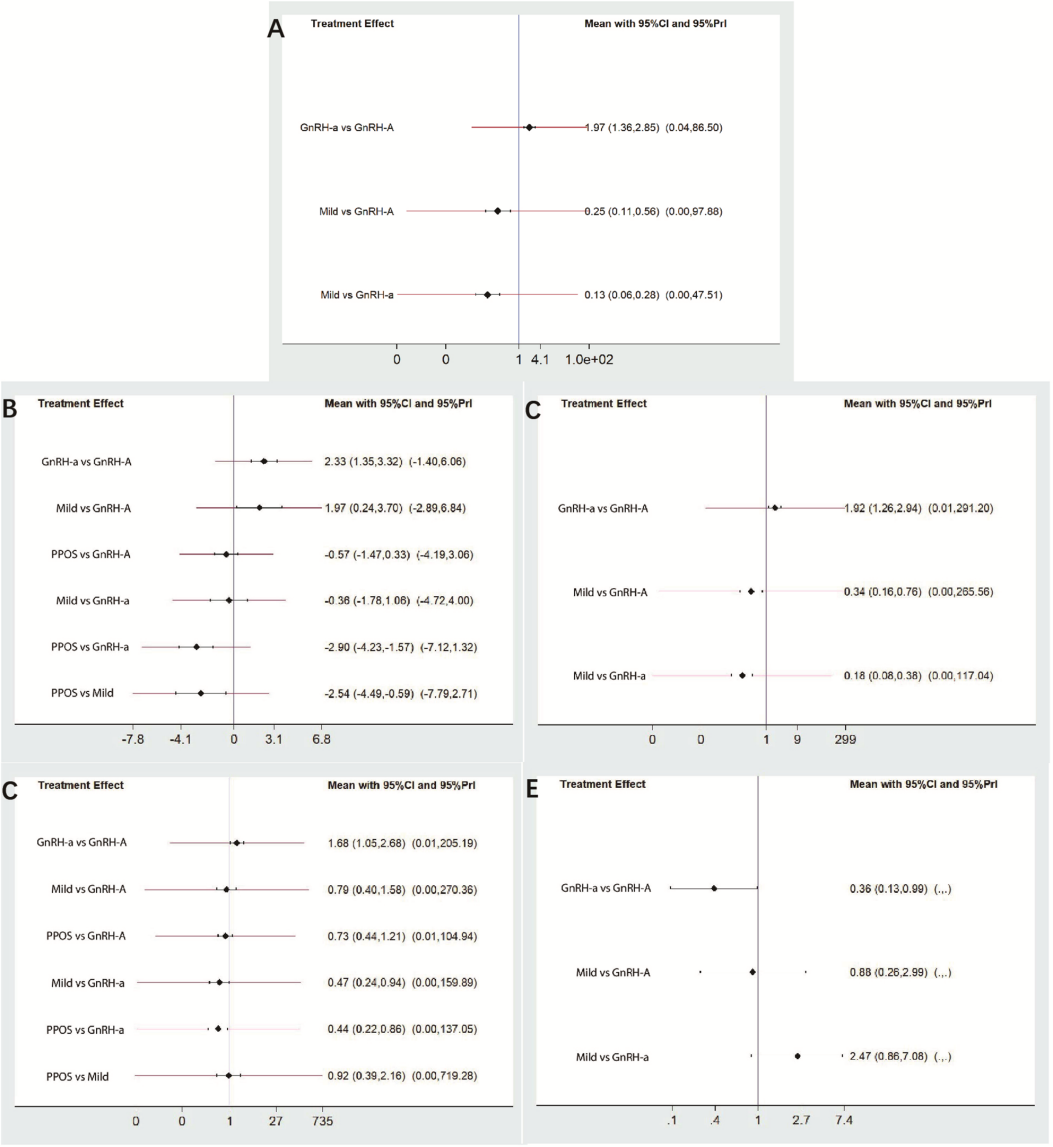
Forest plots of network meta-analyses of the POSEIDON group 3. **A** Live birth rate; **(B)** The number of oocytes retrieved; **(C)** Clinical pregnancy rate; **(D)** Cumulative Live birth rate; **(E)** Cycle cancellation rate

In terms of the live birth rate, GnRH agonist ranked first, demonstrating a significantly higher live birth rate compared to both GnRH antagonist (OR 1.97, 95% CI: 1.36–2.85) and mild stimulation (OR 7.69, 95% CI: 3.57–16.67) (Fig. 3A).

Additionally, GnRH antagonist was more effective than mild stimulation (OR 4.00, 95% CI: 1.79–9.09). The SUCRA rankings for live birth rate were 100.0% for GnRH agonist, 50.0% for GnRH antagonist, and 0.0% for mild stimulation (Fig. S3). When examining oocyte retrieval, both GnRH agonist and mild stimulation resulted in significantly higher oocyte counts compared to GnRH antagonist and PPOS (Fig. 3B). The SUCRA rankings for this outcome were: GnRH agonist (89.5%), mild stimulation (76.6%), GnRH antagonist (29.9%), and PPOS (4.0%) (Fig. S3). For cumulative live birth rate, GnRH agonist demonstrated significantly better outcomes than GnRH antagonist (OR 1.68, 95% CI: 1.05–2.68), mild stimulation (OR 2.13, 95% CI: 1.06–4.17), and PPOS (OR 2.27, 95% CI: 1.16–4.55) (Fig. 3D). The SUCRA rankings for cumulative live birth rate were: GnRH agonist (98.9%), GnRH antagonist (54.6%), mild stimulation (28.1%), and PPOS (18.4%) (Fig. S3). GnRH agonist also led in clinical pregnancy rate, with significantly higher success rates compared to GnRH antagonist (OR 1.92, 95% CI: 1.26–2.94) and mild stimulation (OR 5.56, 95% CI: 2.63–12.5) (Fig. 3C). Moreover, GnRH antagonist was more effective than mild stimulation (OR 2.94, 95% CI: 1.32–6.25). The SUCRA rankings were 100.0% for GnRH agonist, 50.0% for GnRH antagonist, and 0.0% for mild stimulation. Lastly, GnRH agonist was more effective than GnRH antagonist in reducing the risk of cycle cancellation (OR 0.36, 95% CI: 0.13–0.99) (Fig. 3E). The SUCRA values for the risk of cycle cancellation were 78.1% for GnRH antagonist, 68.5% for mild stimulation, and 3.4% for GnRH agonist (Fig. S3).

### POSEIDON group 4

**Fig. 4:**
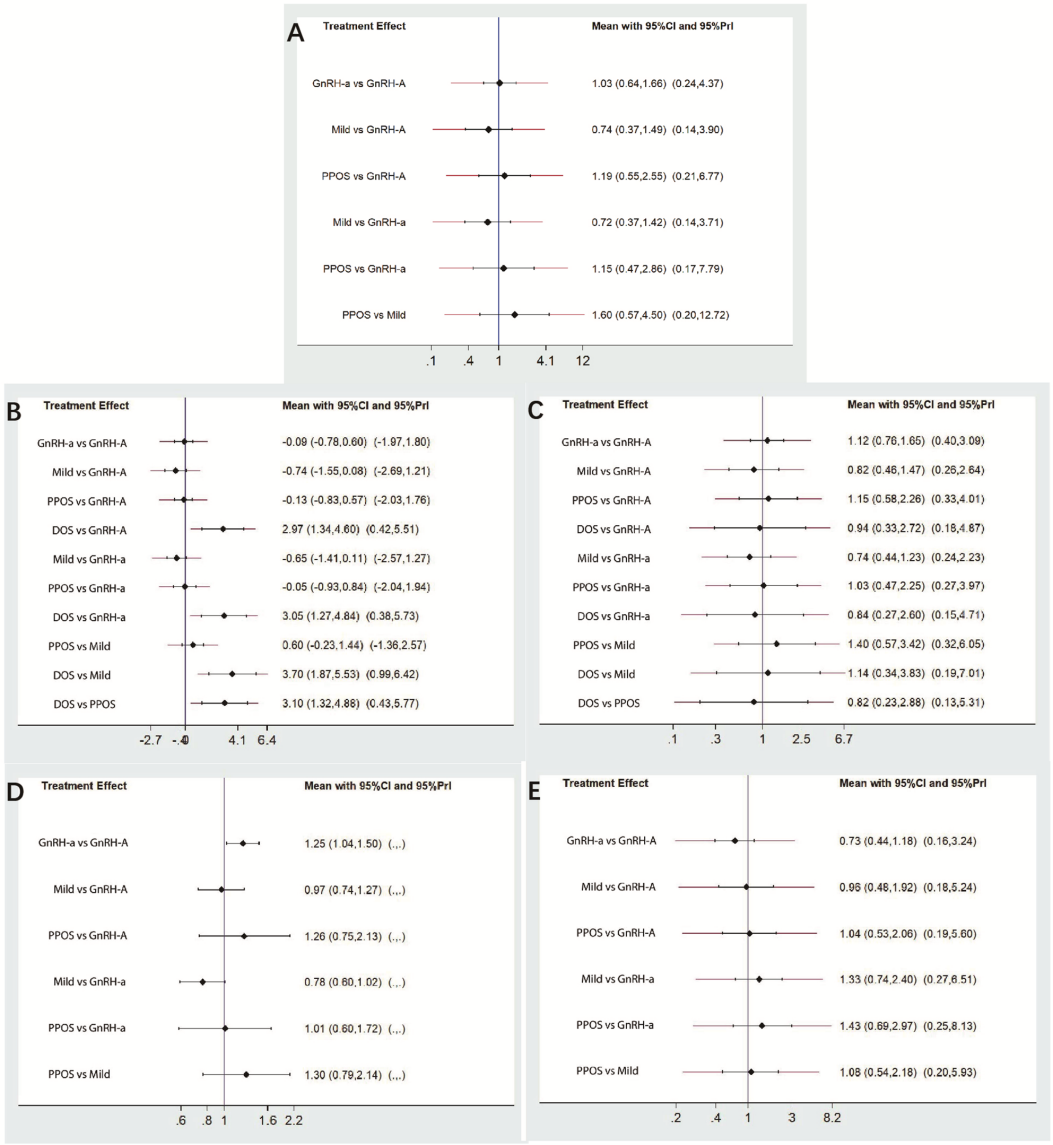
Forest plots of network meta-analyses of the POSEIDON group 4. **A** Live birth rate; **(B)** The number of oocytes retrieved; **(C)** Clinical pregnancy rate; **(D)** Cumulative Live birth rate; **(E)** Cycle cancellation rate

For live birth rate, no significant differences were found between PPOS, GnRH agonist, GnRH antagonist, and mild stimulation (Fig. 4A). The SUCRA rankings were 70.4% for PPOS, 58.3% for GnRH agonist, 52.1% for GnRH antagonist, and 19% for mild stimulation (Fig. S3). Regarding oocyte retrieval, DOS showed the greatest effectiveness, with significant advantages over GnRH antagonist (OR 2.97, 95% CI: 1.34–4.60), GnRH agonist (OR 3.05, 95% CI: 1.27–4.84), PPOS (OR 3.10, 95% CI: 1.32–4.88), and mild stimulation (OR 3.70, 95% CI: 1.87–5.53) (Fig. 4B).

The SUCRA rankings for oocyte retrieval were: DOS (100%), GnRH antagonist (55.2%), GnRH agonist (47.4%), PPOS (44.3%), and mild stimulation (4.2%) (Fig. S3). There were no significant differences observed in clinical pregnancy rates between the various groups, including GnRH agonist, PPOS, GnRH antagonist, DOS, and mild stimulation (Fig. 4C). The SUCRA rankings for clinical pregnancy rate were 67.1% for GnRH agonist, 64.8% for PPOS, 48.0% for GnRH antagonist, 44.3% for DOS, and 25.8% for mild stimulation (Fig. S3). When considering cumulative live birth rate, GnRH agonist proved significantly more effective than GnRH antagonist (OR 1.25, 95% CI: 1.04–1.50) (Fig. 4D). The SUCRA rankings for cumulative live birth rate were: GnRH agonist (81.5%), PPOS (71.9%), GnRH antagonist (26%), and mild stimulation (20.5%) (Fig. S3). Finally, no significant differences in the risk of cycle cancellation were found between PPOS, GnRH antagonist, mild stimulation, and GnRH agonist (Fig. 4E). The SUCRA rankings for cycle cancellation were: PPOS (65.2%), GnRH antagonist (63.5%), mild stimulation (56.6%), and GnRH agonist (14.7%) (Fig. S3).

## Discussion

Our network meta-analysis found that, under the Bologna criteria, the DOS protocol had the most favorable outcomes in oocyte retrieval, clinical pregnancy, live birth and cycle cancellation rates. In contrast, within POSEIDON group 3, the GnRH agonist protocol was superior to others in oocyte retrieval, clinical pregnancy rate, live birth rate, cumulative live birth rate and cycle cancellation rate. In POSEIDON group 4, however, no significant differences were observed among the various ovarian stimulation protocols regarding live birth rate, clinical pregnancy rate, and cycle cancellation. While DOS resulted in higher oocyte retrieval than other protocols, GnRH agonist showed a higher cumulative live birth rate than GnRH antagonist.

These findings may provide valuable references for clinical decision-making in managing patients with low ovarian reserve.

In our network meta-analysis, the DOS protocol emerged as a promising strategy for DOR patients meeting the Bologna criteria, demonstrating superior oocyte retrieval numbers, clinical pregnancy and live birth rates compared with other ovulation induction regimens. The DOS protocol, combining conventional follicular phase stimulation with luteal phases stimulation during the same cycle, aims to maximize the number of oocytes and embryos per cycle [43]. A large RCT aligned with the Bologna criteria reported a live birth rate per cycle of 11% with the GnRH agonist protocol [44]. In contrast, two trials using DOS achieved ongoing pregnancy rates above 20% per cycle in patients with poor prognosis [45, 46]. Moreover, a multicenter study showed that the rate of patients obtaining at least one euploid blastocyst increased significantly from 42.3% after follicular phase stimulation to 65.5% with DOS [46]. These findings align with the POSEIDON concept, which emphasizes increasing the likelihood of obtaining at least one euploid embryo in per patient [47].

However, the mandatory freezing and thawing processes inherent in DOS might introduce additional manipulations and potential risks of oocyte/embryo wastage [7].

Moreover, multiple retrievals can lead to embryo-endometrium asynchrony, adversely affecting implantation and pregnancy outcomes. A recent multicenter, prospective, open, randomized controlled trial comparing the number of oocytes retrieved during a double stimulation cycle with two consecutive GnRH-A protocol cycles in 88 poor responders under Bologna criteria showed no benefit in total oocytes retrieved and mature oocyte count, and increased time to live birth [7]. Therefore, the protocol needs to be carefully considered due to the potential risks and negative impacts of DOS.

Our meta-analysis found that the DOS protocol may improve assisted reproductive outcomes for patients meeting the Bologna criteria. A recent systematic review published by Conforti et al. [48] also underscored the potential of DOS to improve pregnancy outcomes in women with DOR. However, we did not observe this effect in the POSEIDON Group 4 population. Our speculation is that the advantage of the DOS regimen in the Bologna group might be more pronounced in younger DOR patients. Although the Bologna criteria initially defined the POR population, their main limitation lies in the high heterogeneity of the included population. Since the Bologna criteria do not stratify patients by age, the corresponding DOR population may include both younger and older individuals. In contrast, POSEIDON Group 4 is composed exclusively of patients of advanced age with diminished ovarian reserve. This distinction suggests that the advantage of the DOS protocol observed in Bologna-defined populations may be driven primarily by younger DOR patients. We propose that the clinical benefit of the DOS protocol in younger DOR patients may be attributable to several interrelated factors: relatively better oocyte quality and an enhanced capacity to recruit competent follicles within a condensed timeframe. Moreover, due to the limited number of clinical studies involving DOS in POSEIDON-classified populations, data specific to POSEIDON Group 3 patients were not available for inclusion in this analysis. Accordingly, the findings of this study not only demonstrate the significance of age stratification emphasized by the POSEIDON criteria but also highlight the necessity for future RCT to investigate whether patients in POSEIDON Group 3 may benefit from the DOS protocol.

In POSEIDON group 3, the GnRH agonist protocol demonstrated superior outcomes, with significantly higher oocyte retrieval, clinical pregnancy rates, live birth rates, and cumulative live birth rates compared to mild stimulation and GnRH antagonist protocols. This suggests that patients in POSEIDON group 3 may benefit from an increased dose of gonadotropins (Gn). Interestingly, while the 2020 ESHRE guidelines indicated no clear superiority in safety or efficacy between GnRH antagonist and agonist for expected poor responders [49], our study revealed that the GnRH agonist protocol was associated with higher clinical pregnancy rates compared to GnRH antagonists. This advantage may be partly due to the potential negative impact of GnRH antagonist on endometrial receptivity, as evidenced by reduced HOXA10 expression and premature endometrial maturation [50]. Additionally, GnRH agonist had a significantly lower cycle cancellation rate than the GnRH antagonist protocol, further supporting its preferential use in this subgroup.

Conversely, for POSEIDON group 4, our results showed no significant differences in oocyte retrieval, clinical pregnancy rates, or live birth rates among GnRH agonist, GnRH antagonist, PPOS, and mild stimulation protocols. Given the limited number of oocytes and reduced rate of euploid embryos in POSEIDON group 4, the choice of ovarian stimulation protocol appeared to have minimal impact on reproductive outcomes. For patients with advanced age and DOR, high-dose ovarian stimulation protocols may not offer additional benefits, as these patients typically have fewer recruitable follicles. Gonadotropins, regardless of dosage, only support the cohort of follicles sensitive to stimulation without generating new follicles. Moreover, increasing FSH dosages may negatively affect oocyte and embryo quality while raising treatment costs [51, 52]. Recent meta-analyses suggest that low-dose Gn stimulation (Gn≤150IU/d) or Gn combined with letrozole/clomiphene may be equally effective in terms of pregnancy outcomes compared to high-dose stimulation for poor responders [53, 54]. Given these insights, mild stimulation protocols, which offer comparable outcomes at a lower cost, may be a more favorable option for this patient population.

This study is the first network meta-analysis to assess clinical outcomes in DOR patients according to the POSEIDON criteria, comparing various ovarian stimulation protocols. Although studies in POSEIDON groups 3 and 4 are emerging, few comparing different protocols for this population. We standardized patient classification by age, AMH, and AFC and used the 90th percentile of age distribution to enrich subgroup analysis for POSEIDON groups 3 and 4. Several study limitations must be acknowledged. First, we included non-RCTs due to the limited number of studies strictly adhering to the POSEIDON criteria. While this allowed us to broaden the evidence base, it introduced potential bias and reduced the internal validity of our findings. Second, significant variations existed among the five stimulation protocols, such as drug types, gonadotropin doses, treatment durations, and pre-treatments, but limited data prevented subgroup analyses to address clinical heterogeneity. Third, some studies lacked data for POSEIDON groups 3 and 4, limiting comprehensive assessments across protocols. Lastly, many studies did not report the effects of ovarian stimulation on newborn development and health.

## Conclusion

Our findings indicate that the POSEIDON criteria offer a more refined and clinically actionable framework for selecting ovarian stimulation protocols, whereas Bologna criteria continue to be widely employed in earlier studies owing to their historical continuity and the need for comparability with previous research. Our investigation has also yielded some clinically significant findings, most notably that the GnRH agonist protocol demonstrates superior efficacy in POSEIDON Group 3, while no single protocol proves to be superior in POSEIDON Group 4, highlighting the limited benefit of high-dose stimulation in this group. These insights provide an evidence-based rationale for tailoring treatment strategies to patient subtypes and could contribute meaningfully to clinical decision-making in reproductive medicine. Despite the continued application of the Bologna criteria in a large body of clinical research, our investigation highlights that the POSEIDON stratification framework enables more precise protocol for DOR patients, thereby underscoring the imperative for more high-quality randomized controlled trials specifically designed under POSEIDON parameters to establish evidence-based guidelines for ovarian stimulation optimization in DOR populations.

## Supplementary Information

Supplementary Material 1.

## Data Availability

No datasets were generated or analysed during the current study.

## References

[1] Lin HT, Wu MH, Tsai LC, et al. Co-administration of clomiphene citrate and letrozole in mild ovarian stimulation versus conventional controlled ovarian stimulation among POSEIDON group 4 patients [J]. Front Endocrinol (Lausanne). 2021;12:780392. 10.3389/fendo.2021.780392. 35095758 10.3389/fendo.2021.780392 PMC8796317

[2] Kuang Y, Chen Q, Fu Y, et al. Medroxyprogesterone acetate is an effective oral alternative for preventing premature luteinizing hormone surges in women undergoing controlled ovarian hyperstimulation for in vitro fertilization [J]. Fertil Steril. 2015;104(1):62–e7063. 10.1016/j.fertnstert.2015.03.022. 25956370 10.1016/j.fertnstert.2015.03.022

[3] Ubaldi FM, Capalbo A, Vaiarelli A, et al. Follicular versus luteal phase ovarian stimulation during the same menstrual cycle (DuoStim) in a reduced ovarian reserve population results in a similar euploid blastocyst formation rate: new insight in ovarian reserve exploitation [J]. Fertil Steril. 2016;105(6):1488–e14951481. 10.1016/j.fertnstert.2016.03.002. 27020168 10.1016/j.fertnstert.2016.03.002

[4] Ferraretti AP, La Marca A, Fauser B, et al. ESHRE consensus on the definition of ‘poor response’ to ovarian stimulation for in vitro fertilization: the Bologna criteria [J]. Hum Reprod. 2011;26(7):1616–24. 10.1093/humrep/der092. 21505041 10.1093/humrep/der092

[5] Alviggi C, Andersen CY, Buehler K, et al. A new more detailed stratification of low responders to ovarian stimulation: from a poor ovarian response to a low prognosis concept [J]. Fertil Steril. 2016;105(6):1452–3. 10.1016/j.fertnstert.2016.02.005. 26921622 10.1016/j.fertnstert.2016.02.005

[6] Lin G, Zhong X, Li S, et al. The clinical value of progestin-primed ovarian stimulation protocol for women with diminished ovarian reserve undergoing IVF/ICSI: a systematic review and meta-analysis [J]. Front Endocrinol (Lausanne). 2023;14:1232935. 10.3389/fendo.2023.1232935. 37670890 10.3389/fendo.2023.1232935 PMC10476097

[7] Massin N, Abdennebi I, Porcu-Buisson G, et al. The BISTIM study: a randomized controlled trial comparing dual ovarian stimulation (duostim) with two conventional ovarian stimulations in poor ovarian responders undergoing IVF [J]. Hum Reprod. 2023;38(5):927–37. 10.1093/humrep/dead038. 36864699 10.1093/humrep/dead038 PMC10152167

[8] Sfakianoudis K, Pantos K, Grigoriadis S, et al. What is the true place of a double stimulation and double oocyte retrieval in the same cycle for patients diagnosed with poor ovarian reserve? A systematic review including a meta-analytical approach [J]. J Assist Reprod Genet. 2020;37(1):181–204. 10.1007/s10815-019-01638-z. 31797242 10.1007/s10815-019-01638-z PMC7000611

[9] Song D, Shi Y, Zhong Y, et al. Efficiency of mild ovarian stimulation with clomiphene on poor ovarian responders during IVF\ICSI procedures: a meta-analysis [J]. Eur J Obstet Gynecol Reprod Biol. 2016;204:36–43. 10.1016/j.ejogrb.2016.07.498. 27521596 10.1016/j.ejogrb.2016.07.498

[10] Liu XP, Li TT, Wang B, et al. Mild stimulation protocol vs conventional controlled ovarian stimulation protocol in poor ovarian response patients: a prospective randomized controlled trial [J]. Arch Gynecol Obstet. 2020;301(5):1331–9. 10.1007/s00404-020-05513-6. 32211953 10.1007/s00404-020-05513-6

[11] Hutton B, Salanti G, Caldwell DM, et al. The PRISMA extension statement for reporting of systematic reviews incorporating network meta-analyses of health care interventions: checklist and explanations [J]. Ann Intern Med. 2015;162(11):777–84. 10.7326/m14-2385. 26030634 10.7326/M14-2385

[12] Luo D, Wan X, Liu J, et al. Optimally estimating the sample mean from the sample size, median, mid-range, and/or mid-quartile range [J]. Stat Methods Med Res. 2018;27(6):1785–805. 10.1177/0962280216669183. 27683581 10.1177/0962280216669183

[13] Salanti G, Ades AE, Ioannidis JP. Graphical methods and numerical summaries for presenting results from multiple-treatment meta-analysis: an overview and tutorial [J]. J Clin Epidemiol. 2011;64(2):163–71. 10.1016/j.jclinepi.2010.03.016. 20688472 10.1016/j.jclinepi.2010.03.016

[14] Duval S, Tweedie R. Trim and fill: A simple funnel-plot-based method of testing and adjusting for publication bias in meta-analysis [J]. Biometrics. 2000;56(2):455–63. 10.1111/j.0006-341x.2000.00455.x. 10877304 10.1111/j.0006-341x.2000.00455.x

[15] Al-Jeborry MM, Alizzi FJ, Al-Anbari LA. A comparison of 3 different controlled ovarian stimulation protocols in poor women responders chosen according to poseidon criteria: micro-dose, standard flare-up, and antagonist protocol [J]. Int J women’s health Reprod Sci. 2020;8(2):147–52. 10.15296/ijwhr.2020.23.

[16] Youssef MA, Van Wely M, Al-Inany H, et al. A mild ovarian stimulation strategy in women with poor ovarian reserve undergoing IVF: a multicenter randomized non-inferiority trial [J]. Hum Reprod. 2017;32(1):112–8. 10.1093/humrep/dew282. 27836979 10.1093/humrep/dew282

[17] Yu R, Jin H, Huang XF, et al. Comparison of modified agonist, mild-stimulation and antagonist protocols for in vitro fertilization in patients with diminished ovarian reserve [J]. J Int Med Res. 2018;46(6):2327–37. 10.1177/0300060518770346. 29695208 10.1177/0300060518770346 PMC6023056

[18] Davar R, Neghab N, Naghshineh E. Pregnancy outcome in delayed start antagonist versus microdose flare GnRH agonist protocol in poor responders undergoing IVF/ICSI: an RCT [J]. Int J reproductive Biomed. 2018;16(4):255–60. PMC6004594 29942933

[19] Devesa M, Martinez F, Coroleu B, et al. Poor prognosis for ovarian response to stimulation: results of a randomised trial comparing the flare-up GnRH agonist protocol vs. the antagonist protocol [J]. Gynecol Endocrinol. 2010;26(7):509–15. 10.3109/09513591003632191. 20196635 10.3109/09513591003632191

[20] Revelli A, Chiado A, Dalmasso P, et al. Mild’ vs. ‘long’ protocol for controlled ovarian hyperstimulation in patients with expected poor ovarian responsiveness undergoing in vitro fertilization (IVF): a large prospective randomized trial [J]. J Assist Reprod Genet. 2014;31(7):809–15. 24700398 10.1007/s10815-014-0227-y PMC4096882

[21] Racca A, Rodriguez I, Garcia S, et al. Double versus single stimulation in young low prognosis patients followed by a fresh embryo transfer: a randomized controlled trial (DUOSTIM-fresh) [J]. Hum Reprod. 2024. 10.1093/humrep/deae104. 38845190 10.1093/humrep/deae104

[22] Boudry L, Mateizel I, Wouters K, et al. Does dual oocyte retrieval with continuous FSH administration increase the number of mature oocytes in low responders? An open-label randomized controlled trial [J]. Hum Reprod. 2024;39(3):538–47. 10.1093/humrep/dead276. 38199789 10.1093/humrep/dead276

[23] Saharkhiz N, Salehpoor S, Hosseini S, et al. Comparison *In Vitro* fertilization outcomes between DouStim and minimal stimulation protocols in poor ovarian responders: a randomized clinical trial [J]. Int J Fertil Steril. 2024;18(2):135–9. 10.22074/ijfs.2023.552687.1293. 38368516 10.22074/IJFS.2023.552687.1293 PMC10875315

[24] Cenksoy PO, Ficicioglu C, Kizilkale O, et al. The comparision of effect of microdose GnRH-a flare-up, GnRH antagonist/aromatase inhibitor letrozole and GnRH antagonist/clomiphene citrate protocols on IVF outcomes in poor responder patients [J]. Gynecol Endocrinol. 2014;30(7):485–9. 10.3109/09513590.2014.893571. 24592985 10.3109/09513590.2014.893571

[25] Oral S, Karacan M, Akpak YK, et al. Live birth rate with double ovarian stimulation is superior to follicular phase ovarian stimulation per started cycle in poor ovarian responders [J]. J Obstet Gynecol Res. 2021;47(8):2705–12. 10.1111/jog.14871. 34062624 10.1111/jog.14871

[26] Jin BL, Niu ZH, Xu BF, et al. Comparison of clinical outcomes among dual ovarian stimulation, mild stimulation and luteal phase stimulation protocols in women with poor ovarian response [J]. Gynecol Endocrinol. 2018;34(8):694–7. 10.1080/09513590.2018.1435636. 29409363 10.1080/09513590.2018.1435636

[27] Zhang XY, Feng T, Yang JH, et al A flexible short protocol in women with poor ovarian response over 40 years old [J]. J Ovarian Res. 2021;14(1). 10.1186/s13048-020-00761-1. 10.1186/s13048-020-00761-1 PMC7786950 33402208

[28] Guo Y, Jiang H, Hu S et al. Efficacy of three COS protocols and predictability of AMH and AFC in women with discordant ovarian reserve markers: a retrospective study on 19,239 patients [J]. J Ovarian Res. 2021;14(1). 10.1186/s13048-021-00863-4. 10.1186/s13048-021-00863-4 PMC8403432 34454544

[29] Tu X, You B, Jing M et al. Progestin-primed ovarian stimulation versus mild stimulation protocol in advanced age women with diminished ovarian reserve undergoing their first in vitro fertilization cycle: a retrospective cohort study [J]. Front Endocrinol. 2022;12. 10.3389/fendo.2021.801026. 10.3389/fendo.2021.801026 PMC8818948 35140685

[30] Li Y, Yang W, Chen X, et al. Comparison between follicular stimulation and luteal stimulation protocols with clomiphene and HMG in women with poor ovarian response [J]. Gynecol Endocrinol. 2016;32(1):74–7. 10.3109/09513590.2015.1081683. 26370530 10.3109/09513590.2015.1081683

[31] Du M, Zhang J, Li Z et al. Comparison of the cumulative live birth rates of progestin-primed ovarian stimulation and flexible gnrh antagonist protocols in patients with low prognosis [J]. Front Endocrinol. 2021;12 10.3389/fendo.2021.705264. 10.3389/fendo.2021.705264 PMC8475782 34589055

[32] Li F, Ye T, Kong H, et al. Efficacies of different ovarian hyperstimulation protocols in poor ovarian responders classified by the POSEIDON criteria [J]. Aging. 2020;12(10):9354–64. 10.18632/aging.103210. 32470947 10.18632/aging.103210 PMC7288941

[33] Huang PX, Tang ML, Qin AP. Progestin-primed ovarian stimulation is a feasible method for poor ovarian responders undergoing in IVF/ICSI compared to a GnRH antagonist protocol: A retrospective study [J]. J Gynecol Obstet Hum Reprod. 2019;48(2):99–102. 10.1016/j.jogoh.2018.10.008. 30321608 10.1016/j.jogoh.2018.10.008

[34] Hu L, Bu Z, Guo Y, et al. Comparison of different ovarian hyperstimulation protocols efficacy in poor ovarian responders according to the bologna criteria [J]. Int J Clin Exp Med. 2014;7(4):1128–doi1134. 24955194 PMC4057873

[35] Zhao S, Wang C. Efficacy of progestin-primed ovarian stimulation (PPOS) versus minimal stimulation in women of advanced maternal age with poor ovarian response under the Patient-Oriented Strategies Encompassing Individualized Oocyte Number (POSEIDON) criteria [J]. Ann Palliat Med. 2023;12(1):133–40. 10.21037/apm-22-1448. 36747387 10.21037/apm-22-1448

[36] Li Z, Jia RL, Wang KX et al. Analysis of cumulative live birth rate and perinatal outcomes in young patients with low anti-mullerian hormone levels using two ovulation promotion protocols: A cohort study [J]. Front Endocrinol. 2022;13. 10.3389/fendo.2022.938500. 10.3389/fendo.2022.938500 PMC9389309 35992097

[37] Li W, Zhang W, Zhao H, et al. Efficacy of the depot gonadotropin-releasing hormone agonist protocol on in vitro fertilization outcomes in young poor ovarian responders from POSEIDON group 3 [J]. Int J Gynecol Obstet. 2022;157(3):733–40. 10.1002/ijgo.13933. 10.1002/ijgo.13933 34534357

[38] Kao TC, Hsieh YC, Yang IJ, et al. Progestin-primed ovarian stimulation versus GnRH antagonist protocol in poor responders: risk of premature LH surge and outcome of oocyte retrieval [J]. J Formos Med Assoc. 2023;122(1):29–35. 10.1016/j.jfma.2022.08.023. 36123235 10.1016/j.jfma.2022.08.023

[39] Wang M, Li L, Zhu H, et al. Comparison of progestin-primed ovarian stimulation regimen and antagonist regimen in women aged 35 years or older with diminished ovarian reserve: A propensity score-matched study [J]. Int J Gynaecol Obstet. 2024;167(1):162–8. 10.1002/ijgo.15543. 38619107 10.1002/ijgo.15543

[40] Duan XY, Li Z, Li MM, et al. Efficacies of different ovarian hyperstimulation protocols in elderly patients with poor ovarian response [J]. Eur Rev Med Pharmacol Sci. 2023;27(23):11606–13. 10.26355/eurrev_202312_34599. 38095408 10.26355/eurrev_202312_34599

[41] Pal A, Mani T, Chinta P, et al. Effectiveness of GnRH Agonist Short Protocol Versus GnRH Antagonist Protocol in POSEIDON Groups 3 and 4: a Retrospective Cohort Study [J]. Reprod Sci. 2023;30(8):2481–8. 10.1007/s43032-023-01196-x. 36808612 10.1007/s43032-023-01196-x

[42] Al-Hussaini TK, Mohamed AA, Askar A, et al. Ovarian Stimulation in Patient-oriented Strategies Encompassing Individualised Oocyte Number-4 Category; Antagonist versus Short-agonist Protocols [J]. J Hum Reprod Sci. 2023;16(3):212–7. 10.4103/jhrs.jhrs_72_23. 38045497 10.4103/jhrs.jhrs_72_23 PMC10688281

[43] Adams GP, Singh J, Baerwald AR. Large animal models for the study of ovarian follicular dynamics in women [J]. Theriogenology. 2012;78(8):1733–48. 10.1016/j.theriogenology.2012.04.010. 22626769 10.1016/j.theriogenology.2012.04.010

[44] Humaidan P, Chin W, Rogoff D, et al. Efficacy and safety of follitropin alfa/lutropin alfa in ART: a randomized controlled trial in poor ovarian responders [J]. Hum Reprod. 2017;32(7):1537–8. 10.1093/humrep/dex208. 28541398 10.1093/humrep/dex208 PMC5946864

[45] Alsbjerg B, Haahr T, Elbaek HO, et al. Dual stimulation using corifollitropin alfa in 54 Bologna criteria poor ovarian responders - a case series [J]. Reprod Biomed Online. 2019;38(5):677–82. 10.1016/j.rbmo.2019.01.007. 30795977 10.1016/j.rbmo.2019.01.007

[46] Vaiarelli A, Cimadomo D, Trabucco E, et al. Double Stimulation in the Same Ovarian Cycle (DuoStim) to Maximize the Number of Oocytes Retrieved From Poor Prognosis Patients: A Multicenter Experience and SWOT Analysis [J]. Front Endocrinol (Lausanne). 2018;9:317. 10.3389/fendo.2018.00317. 29963011 10.3389/fendo.2018.00317 PMC6010525

[47] Esteves SC, Humaidan P, Alviggi C et al. The novel POSEIDON stratification of ‘Low prognosis patients in assisted reproductive technology’ and its proposed marker of successful outcome [J]. F1000Research. 2016;5. 10.12688/f1000research.10382.1. 10.12688/f1000research.10382.1 PMC5302217 28232864

[48] Conforti A, Carbone L, Di Girolamo R, et al. Therapeutic management in women with a diminished ovarian reserve: a systematic review and meta-analysis of randomized controlled trials [J]. Fertil Steril. 2025;123(3):457–76. 10.1016/j.fertnstert.2024.09.038. 39332623 10.1016/j.fertnstert.2024.09.038

[49] Ovarian Stimulation T, Bosch E, Broer S et al. ESHRE guideline: ovarian stimulation for IVF/ICSI(†) [J]. Hum Reprod Open, 2020, 2020(2):hoaa009. 10.1093/hropen/hoaa009. 10.1093/hropen/hoaa009 PMC7203749 32395637

[50] Ren J, Sha A, Han D, et al. Does prolonged pituitary down-regulation with gonadotropin-releasing hormone agonist improve the live-birth rate in in vitro fertilization treatment? [J]. Fertil Steril. 2014;102(1):75–81. 10.1016/j.fertnstert.2014.03.030. 24746740 10.1016/j.fertnstert.2014.03.030

[51] Boudry L, Racca A, Tournaye H, et al. Type and dose of gonadotropins in poor ovarian responders: does it matter? [J]. Ther Adv Reprod Health. 2021;15:26334941211024203. 10.1177/26334941211024203. 34263173 10.1177/26334941211024203 PMC8243085

[52] Bastu E, Buyru F, Ozsurmeli M, et al. A randomized, single-blind, prospective trial comparing three different gonadotropin doses with or without addition of letrozole during ovulation stimulation in patients with poor ovarian response [J]. Eur J Obstet Gynecol Reprod Biol. 2016;203:30–4. 10.1016/j.ejogrb.2016.05.027. 27236602 10.1016/j.ejogrb.2016.05.027

[53] Datta AK, Maheshwari A, Felix N, et al. Mild versus conventional ovarian stimulation for IVF in poor responders: a systematic review and meta-analysis [J]. Reprod Biomed Online. 2020;41(2):225–38. 10.1016/j.rbmo.2020.03.005. 32546333 10.1016/j.rbmo.2020.03.005

[54] Youssef MA, Van Wely M, Mochtar M, et al. Low dosing of gonadotropins in in vitro fertilization cycles for women with poor ovarian reserve: systematic review and meta-analysis [J]. Fertil Steril. 2018;109(2):289–301. 10.1016/j.fertnstert.2017.10.033. 29317127 10.1016/j.fertnstert.2017.10.033

