# Supplementary material for: Optimal selection of ovarian stimulation protocol for infertile women with diminished ovarian reserve based on Bologna and POSEIDON criteria: a network meta-analysis
Source: Reprod Biol Endocrinol. 2026 Jun 5;24:76. doi: 10.1186/s12958-026-01573-6 (PMC13459166; doi:10.1186/s12958-026-01573-6)
Supplement: Supplementary file 1 [file 12958_2026_1573_MOESM1_ESM.pdf]

Supplementary Materials

Table legends

Table S1. Search strategy.

Table S2. Characteristics of the included studies.

Table S3. Assessment of risk bias for retrospective cohort studies.

Table S4. PRISMA 2020 checklist.

Figure legends

Figure S1. Risk of bias across through all studies included RCTs.

Figure S2. The network plots for the primary and secondary outcomes.

Figure S3. Surface under the cumulative ranking curve for the secondary outcomes.

Figure S4. Comparison-adjusted funnel plot for the primary and secondary outcomes.

Table S1. Search strategy.

**Pubmed**
#1 “diminished ovarian reserve” or “DOR” or “poor ovarian response” or “POR” or “POSEIDON criteria” or “Bologna criteria” or “ovarian reserve” or “anti-Müllerian hormone” or “AMH” or “antral follicle count” or “AFC”<br>#2 “ovulation induction” OR “ovarian stimulation” OR “controlled ovarian stimulation” OR “COS” OR “Controlled ovarian hyperstimulation” OR “COH”<br>#3 “in vitro fertilization outcome” OR “IVF outcome” OR “intracytoplasmic sperm injection outcome” OR “ICSI outcome” OR “IVF/ICSI outcome” OR “ART outcome”<br>#4 #1 AND #2 AND #3
**EMBASE**
#1 diminished AND ovarian AND reserve OR 'dor' OR (poor AND ovarian AND response) OR 'por' OR (poseidon AND criteria) OR (ovarian AND reserve) OR ('anti müllerian' AND hormone) OR 'amh' OR (antral AND follicle AND count) OR 'afc'<br>#2 in AND vitro AND fertilization AND outcome* OR (ivf AND outcome*) OR (intracytoplasmic AND sperm AND injection AND outcome*) OR (icsi AND outcome*) OR (art AND outcome*) OR (fet AND outcome*)<br>#3 ovulation AND induc* OR (ovarian AND stimulat*) OR (controlled AND ovarian AND stimulation) OR 'cos' OR (controlled AND ovarian AND hyperstimulation) OR 'coh'<br>#4 #1 AND #2 AND #3
**WEB OF SCIENCE**

#1 ovulation induc* (All Fields) or ovarian stimulat* (All Fields) or controlled ovarian stimulation (All Fields) or COS (All Fields) or Controlled ovarian hyperstimulation (All Fields) or COH (All Fields)<br>#2 ((((((((ALL=(diminished ovarian reserve)) OR ALL=(DOR)) OR ALL=(poor ovarian response)) OR ALL=(POR)) OR ALL=(POSEIDON criteria)) OR ALL=(ovarian reserve)) OR ALL=(anti-Müllerian hormone)) OR ALL=(AMH)) OR ALL=(antral follicle count)) OR ALL=(AFC)<br>#3 in vitro fertilization outcome* (All Fields) or IVF outcome* (All Fields) or intracytoplasmic sperm injection outcome* (All Fields) or ICSI outcome* (All Fields) or ART outcome* (All Fields) or FET outcome* (All Fields)<br>#4 #1 AND #2 AND #3
**Cochrane library search strategy Database**
#1 (diminished ovarian reserve or DOR or poor ovarian response or POR or POSEIDON criteria or ovarian reserve or anti-Müllerian hormone or AMH or antral follicle count or AFC) (Word variations have been searched)(Word variations have been searched)S Limits<br>#2 (ovulation induc* or ovarian stimulat* or controlled ovarian stimulation or COS or COH or Controlled ovarian hyperstimulation) (Word variations have been searched)<br>(Word variations have been searched)S Limits<br>#3 (in vitro fertilization outcome* or IVF outcome* or intracytoplasmic sperm injection outcome* or ICSI outcome* or ART outcome* or FET outcome*) (Word variations have been searched)<br>(Word variations have been searched)S Limits<br>#4 #1 and #2 and #3

Table S2. Characteristics of the included studies.

	Study	Country	Design	Participants	Stimulation protocol					Outcomes
					Intervention	n	Age	AMH	AFC	
**Bologna group**										
1	Al-Jeborry et al, 2020	Iraq	RCT	POSEIDON criteria	**GnRH agonist (1):** triptorelin 25 µg/d + FSH 300 IU/d + HMG 150 U/d	38	36.6 ± 4.2	0.56 ± 0.24	4.73 ± 1.36	c-e
					**GnRH agonist (2):** triptorelin 100 µg/d + high dose Gn	38	37.6 ± 4.7	0.57 ± 0.34	4.92 ± 1.93	
					**GnRH antagonist:** high dose Gn + Cetrotide 0.25 mg/d	38	35.6 ± 5.8	0.56 ± 0.28	4.73 ± 1.36	
2	Cenksoy et al, 2014	Turkey	non-RCT	AMH < 1.1 ng/ml	**GnRH agonist:** leuprolide acetate 40 µg/bid + rFSH 450 IU/d	83	38.2 ± 4.7	0.43 ± 0.44	NR	c-e
					**GnRH antagonist (1):** LE 2.5 mg/d + rFSH 450 IU/d + cetrorelix 0.25 mg/d	70	36.7 ± 5.1	0.47 ± 0.5	NR	
					**GnRH antagonist (2):** CC 100 mg/d + rFSH 450 IU/d + cetrorelix 0.25 mg/d	72	36.8 ± 5.6	0.34 ± 0.28	NR	
3	Oral et al., 2021	Turkey	non-RCT	Bologna criteria	**GnRH antagonist:** rFSH 225 IU/d + HMG 75 IU/d + Cetrotide 0.25 mg	146	36.6 ± 4.4	0.7 ± 0.3	3.8 ± 1.3	a,c-e
					**DOS:** FPS rFSH 225 IU/d + HMG 75 IU/d + Cetrotide 0.25 mg; LPS rFSH 225 IU + HMG 75 IU	58	36.0 ± 4.1	0.7 ± 0.4	4.1 ± 2.4	
4	Youssef et al., 2017	Egypt	RCT	age ≥ 35 years or FSH >10 IU/ml; AFC < 5 or POR or cycle cancellation during a previous cycle	**GnRH antagonist:** FSH 150 IU + Cetrotide 0.25 mg/d	187	36.5 ± 3.9	0.5 ± 0.6	5.3 ± 1.6	d,e
					**GnRH agonist:** Decapepetyl 0.1 mg + HMG 450 IU	196	36.6 ± 4.3	0.6 ± 0.6	6.4 ± 2.9	
5	Yu et al, 2018	China	RCT	DOR: age < 42 ; FSH > 15.0 IU/l, or the ratio of FSH to LH > 3; AFC ≤ 8； AMH< 1.5	**GnRH agonist:** triptorelin 3.75 mg + HMG 225-300 IU	54	35.4 ± 3.8	0.86 ± 0.31	5.64 ± 1.39	a,c-e
					**GnRH antagonist (1):** LE 5 mg + HMG 75 IU + cetrorelix 0.25 mg	52	37.3 ± 4.6	0.79 ± 0.28	5.44 ± 1.90	
					**GnRH antagonist (2):** HMG 150 IU (adjusted by 75 IU more or less per day) + cetrorelix 0.25 mg	60	36.7 ± 3.6	0.85 ± 0.42	6.23 ± 2.64	
6	Boudry et al, 2024	Belgium	RCT	DOR	**GnRH antagonist:** FA 150ug + rFSH 225 IU + Ganirelix	24	36.3 (3.6)	0.5 (0.2)	6.6 (3.3)	b,d
					**DOS:** LPS CFA 150 ug + rFSH 225 IU + Ganirelix; FPS: rFSH 225 IU + Ganirelix	24	35.6 (2.9)	0.6 (0.3)	5.7 (2.8)	
7	Jin et al, 2018	China	non-RCT	Bologna criteria	**DOS:** LPS CC 50 or 100 mg + HMG 150–300 IU; FPS CC 50 or 100 mg or LE 5 mg + HMG 150–300 IU + Cetrotide 0.125 mg	76	36.9 ± 6.2	NR	4.2 ± 1.1	c-e
					**Mild stimulation:** CC 50 or 100 mg + HMG 150–300 IU	52	37.0 ± 5.2	NR	4.1 ± 1.2	
					**GnRH antagonist:** CC 50 or 100 mg or LE 5 mg + HMG 150–300 IU + Cetrotide 0.125 mg	132	37.6 ± 5.7	NR	4.0 ± 1.3	
8	Liu et al, 2020	China	RCT	Bologna criteria	**GnRH antagonist:** LE 5 mg + rFSH 150 IU + Cetrotide 0.25 mg	97	37 (34 - 41)	0.6 (0.4 - 1.0)	4 (4 - 6)	a-e
					**GnRH agonist:** Triptorelin 0.05 mg + rFSH 300 IU	94	39 (32 - 41)	0.8 (0.5 - 1.1)	5 (4 - 6)	
9		India	non-RCT	POSEIDON G4 and G3	**GnRH antagonist:** Gn 300-450 IU + Cetrorelix	138	NR	NR	NR	a,c,e

	Atri et al, 2023				**GnRH agonist:** Lupride acetate + Gn	157	NR	NR	NR	
10	Guo et al, 2021(1)	China	non-RCT	AFC ≥ 5 and AMH <1.2 ng/ml	**GnRH antagonist:** rFSH or HMG + Cetrotide 0.25 mg/d	633	33.2 ± 4.9	0.84 ± 0.24	7.07 ± 2.37	a-d
					**GnRH agonist (1):** triptorelin acetate 0.1 mg+ Gn and triptorelin dose was reduced to 0.05 mg/d	116	33.0 ± 4.6	0.93 ± 0.20	9.01 ± 2.64	
					**GnRH agonist (2):** triptorelin 3.75 mg+Gn	86	31.1 ± 4.1	0.92 ± 0.25	10.01 ± 3.7	
11	Guo et al, 2021(1)	China	non-RCT	AFC<5 and AMH ≥1.2 ng/ml	**GnRH antagonist:** rFSH or HMG + Cetrotide 0.25 mg/d	414	33.2 ± 4.8	2.38 ± 1.32	3.32 ± 0.91	a-d
					**GnRH agonist (1):** triptorelin acetate 0.1 mg + Gn and triptorelin dose was reduced to 0.05 mg/d	12	34.1 ± 4.3	4.01 ± 2.45	3.17 ± 1.40	
					**GnRH agonist (2):** triptorelin 3.75 mg + Gn	20	31.8 ± 4.9	3.28 ± 1.75	3.25 ± 1.33	
12	Revelli et al, 2014	Italy	RCT	FSH 10-20 IU/l in the presence of E2 <80 pg/ml; AMH 0.14 - 1.0 ng/ml ; AFC 4 - 10	**GnRH antagonist:** CC 100 mg + Gn 150 IU + Cetrotide 0.25 mg	355	38.4 ± 3.3	0.70 ± 0.39	5.3 ± 2.6	c-e
					**GnRH agonist**: GnRH-a 0.8 mg + Gn 300-400 IU + GnRH-a 0.4 mg	340	37.6 ± 3.6	0.69 ± 0.37	6.1 ± 2.7	
**POSEIDON group 3**										
1	Du et al, 2021	China	non-RCT	POSEIDON 3 criteria	**PPOS:** MPA 6 mg + hMG 150 ~ 300 IU	77	< 35	0.8 ± 0.2	3.0 ± 0.9	b,d
					**GnRH antagonist:** HMG 150 - 300 IU + GnRH-A 0.25 mg	34	< 35	0.8 ± 0.2	3.0 ± 0.9	
2	Li et al, 2020 (2)	China	non-RCT	POSEIDON 3 criteria	**GnRH agonist (1):** Diphereline 3.75 mg + Gn	159	< 35	0.57 ± 0.2	NR	a,c-e
					**GnRH agonist (2):** Decapeptyl 0.1 mg + Gn	124	< 35	0.55 ± 0.3	NR	
					**GnRH antagonist:** Gn 112.5-300 IU + cetrorelix0.25 mg	127	< 35	0.51 ± 0.3	NR	
3	Hu et al, 2014 (2)	China	non-RCT	Bologna criteria and age ≤ 35	**GnRH agonist (1):** triptorelin 0.1 mg + rFSH 150-300 IU + none or triptorelin 0.05 mg	190	NR	5.54 ± 2.57	NA	c-e
					**GnRH agonist (2):** GnRH-a 3.75 mg or GnRH-a 1.875 mg in midluteal phase twice + HMG	48	NR	4.64 ± 2.22	NA	
					**Mild stimulation:** LE + HMG	25	NR	3.7 ± 2.5	NA	
4	Li et al, 2022 (1)	China	non-RCT	age < 35 and AMH < 0.5 ng/mL	**GnRH antagonist:** Stryker or Ganirelix + Gn 150-300 IU	54	≤ 35	< 0.5	6 (4, 7)	b,d
					**PPOS:** Gn 75-300 IU + MPA 6 mg	214	≤ 35	< 0.5	4 (3, 6)	
5	Li et al, 2022 (1)	China	non-RCT	age < 35 and 0.5 ≤ AMH < 1.2 ng/mL	**GnRH antagonist:** Stryker or Ganirelix + Gn 150-300 IU	191	≤ 35	0.5 - 1.2	8 (6, 11)	b,d
					**PPOS:** Gn 75-300 IU + MPA 6 mg	339	≤ 35	0.5 - 1.2	7 (5, 10)	
6	Li et al, 2021	China	non-RCT	POSEIDON group 4 criteria, first IVF cycle and BMI <30	**Mild stimulation:** CC + HMG	186	31.8 ± 2.5	0.72 ± 0.32	5.38 ± 1.52	b,d
					**GnRH antagonist:** rFSH + Ganerik 0.25 mg	145	31.8 ± 2.6	0.71 ± 0.29	5.54 ± 1.90	
					**GnRH agonist:** Decapeptyl 3.75 mg + Gn	120	31.9 ± 2.3	0.72 ± 0.35	5.75 ± 2.45	
**POSEIDON group 4**										

1	Davar et al, 2018	Iran	RCT	Bologna criteria	**GnRH antagonist:** Cetrotide 0.25 mg + FSH 300 IU	50	38.4 ± 2.5	0.75 ± 0.48	4.88 ± 1.99	c,d
					**GnRH agonist:** Buserelin 50ug + FSH 300 IU	50	39.8 ± 2.9	0.93 ± 0.52	4.78 ± 2.37	
2	Zhang et al, 2021	China	non-RCT	POSEIDON criteria and age > 40	**GnRH agonist (1):** Triptorelin 0.05 mg + Gn	237	44.4 ± 3.3	0.53 ± 0.57	4.94 ± 1.99	a,c-e
					**GnRH agonist (2):** Triptorelin 0.1 mg + Gn 150–225 IU	76	44.0 ± 3.4	0.46 ± 0.53	4.81 ± 1.70	
					**Mild stimulation:** LE 2.5-5 mg + HMG or rFSH 75-150 IU	175	44.7 ± 2.5	0.50 ± 0.57	4.85 ± 2.18	
3	Tu et al, 2022	China	non-RCT	Age ≥ 35; DOR (AMH < 0.5 - 1.1 ng/mL or AFC < 5 - 7); first IVF/ICSI procedure	**Mild stimulation:** CC 50-100 mg or LE 2.5-5 mg + Gn 0-225 IU	600	40.0 ± 3.3	0.65 (1.07 - 0.39)	4 (5 - 3)	b,d
					**PPOS:** medroxyprogesterone acetate10 mg or dydrogesterone 20 mg + HMG 150-300 IU/d	139	39.5 ± 3.0	0.78 (1.30 - 0.45)	5 (6 - 3)	
4	Li et al, 2015	China	non-RCT	Bologna criteria	**Mild stimulation:** CC 100 mg + hMG 75–150 IU	33	40.5 ± 4.0	0.5 ± 0.5	5.2 ± 1.9	a,c-e
					**GnRH antagonist:** CC 100 mg + hMG 75–150 IU + Cetrotide 0.25 mg	98	39.3 ± 3.5	0.6 ± 0.5	5.2 ± 2.1	
5	Devesa et al, 2010	Spain	RCT	age ≤ 45 and at least one of the following: prior cycle cancellation; prior poor response, a pathologic CCCT (FSH day 3/ FSH day 10 ≥ 25) and/or AFC ≤ 7 follicles	**GnRH agonist:** leuprolide acetate 0.1 ml + rFSH 375 IU (OR rFSH 300 IU + hMG 75 IU)	80	38.5 + 3.9	NR	5.19 + 2.44	c-e
					**GnRH antagonist:** rFSH 375 IU (OR rFSH 300 IU + hMG 75 IU) + ganirelix	92	38.9 + 3.8	NR	4.67 + 1.86	
6	Du et al, 2021	China	non-RCT	POSEIDON group 4	**PPOS:** MPA 6 mg + hMG 150 - 300 IU	362	≥ 35	0.7 ± 0.2	3.0 ± 1.0	b,d
					**GnRH antagonist:** HMG 150 - 300 IU + GnRH-A 0.25 mg	95	≥ 35	0.7 ± 0.2	3.0 ± 1.0	
7	Li et al, 2020 (1)	China	non-RCT	POSEIDON group 4	**GnRH agonist (1):** Diphereline 3.75 mg + Gn	123	≥ 35	0.54 ± 0.2	NR	a,c-e
					**GnRH agonist (2):** Decapeptyl 0.1 mg + Gn	259	≥ 35	0.54 ± 0.3	NR	
					**GnRH antagonist:** Gn 112.5-300 IU + cetrorelix0.25 mg	457	≥ 35	0.53 ± 0.3	NR	
8	Huang et al, 2019	China	non-RCT	A frozen preservation of two or more embryos, with at least one good-quality embryo, and at least two of the following criteria: age > 40; poor response; AFC < 6	**PPOS:** HMG 150-225 IU + MPA 10 mg	63	41.4 ± 3.6	NR	3.10 ± 1.46	a,c-e
					**GnRH antagonist:** rFSH 150-225 IU/d/5; following,the FSH dose was adjusted according to the ovarian response and E2 level + cetrorelix 0.25 mg	123	41.35 ± 3.56<br>40.70 ± 3.55	NR	3.57 ± 1.65	
9	Hu et al, 2014 (1)	China	non-RCT	Bologna criteria and age ≥ 35	**GnRH agonist (1):** triptorelin 0.1 mg + rFSH 150-300 IU + none or triptorelin 0.05 mg	210	≥35	NR	4.46 ± 2.43	c-e
					**GnRH agonist (2):** GnRH-a 3.75 mg or GnRH-a 1.875 mg in midluteal phase twice + HMG	48	≥35	NR	4.35 ± 2.21	
					**Mild stimulation:** LE + HMG	71	NR	2.5 ± 1.6	NR	

10	Zhao et al, 2023	China	non-RCT	POSEIDON group 4 criteria	**PPOS:** MPA 10 mg + HMG 225 IU	52	40.5 (38, 43)	0.36 (0.13, 0.56)	2.00 (1.00, 4.00)	d,e
					**Mild stimulation:** CC 50 mg + HMG 150–225 IU	50	39 (37, 42)	0.37 (0.12, 0.52)	2.00 (2.00, 4.00)	
11	Wang et al, 2024	China	non-RCT	age ≥ 35 years who met any two of the following criteria: FSH ≥ 10 IU/L, AMH <1.1 ng/mL, AFC<7.	**PPOS:** DYG 10 mg + HMG + FSH	87	39 (37, 42)	0.59 (0.39, 0.87)	5 (4, 6)	a,c-e
					**GnRH antagonist:** FSH + HMG + cetrotide	87	39 (36, 42)	0.61 ± 0.31	5 (3, 6)	
12	Racca et al, 2024	Spain	RCT	POSEIDON group 3 and 4 criteria	**GnRH antagonist:** FSH 250 IU + corifollitoppin α 150ug + GnRH-A	54	37 (35–39)	0.57 (0.37, 0.89)	7 (4, 9)	c,d
					**DOS:** LPS corifollitoppin α 150ug + FSH 250 IU GnRH-A; FPS: FSH 250 IU + corifollitoppin α 150 ug + GnRH-A	53	37 (36–39)	0.55 (0.29, 0.82)	6 (4, 10.5)	
13	Nasrin et al, 2024	?	RCT	age ≥ 35, AFC <5, and AMH <1.2 ng/ml	**DOS:** LPS CFA 150ug + rFSH 225 IU + Ganirelix; FPS: rFSH 225 + Ganirelix	21	39.2 ± 2.4	0.74 ± 0.39	5.36 ± 1.67	d
					**GnRH antagonist:** LE 50Mg + HMG 150 IU + Cetrotide	21	38.5 ± 3.0	0.65 ± 0.35	4.77 ± 1.77	
14	Duan et al, 2023	China	non-RCT	≥35 years; Bologna criteria	**GnRH agonist:** Diphereline 3.75 mg + rFSH 225 IU	1225	39.7 ± 2.6	0.68 ± 0.34	NR	a-d
					**GnRH antagonist:** FSH + GnRH-A	1038	39.8 ± 2.9	0.71 ± 0.51	NA	
					**Mild stimulation:** FSH 75-150 IU	397	39.7 ± 3.0	0.63 ± 0.55	NA	
15	Tarek et al, 2023	Egypt	non-RCT	POSEIDON group 4	**GnRH agonist:** triptorelin 0.1 mg + Gn 300-450 IU	79	38 (3)	0.49 (0.56)	4 (2)	a,c-e
					**GnRH antagonist:** Gn 300-450 IU + cetrorelix 0.25 mg	111	39 (4)	0.39 (0.29)	3 (2)	
16	Kao et al, 2023	Taiwan	non-RCT	Bologna criteria	**PPOS:** FSH/HMG 150-350 IU + MPA 10 mg	54	41.2 (3.4)	0.55 (0.38)	2.7 (1.3)	d
					**GnRH antagonist:** FSH/HMG 150-350 IU + ganirelix 0.25 mg	260	42.0 (2.6)	0.63 (0.37)	3.2 (1.8)	

a. Live birth rate; b. Cumulative live birth rate; c. Clinical pregnancy rate; d. The number of oocytes retrieved; e. Cycle cancellation rate.

Table S3. Assessment of risk bias for retrospective cohort studies.

Cohort studies	Selection				Comparability	Outcome			Total score
	Representativeness of the exposed cohort	Selection of the non-exposed cohort	Ascertainment of exposure	Outcome of interest was not present at the start of the study	Comparability of cochorts on the basis of the design or analysis	Assessment of outcome	Was follow up long enough for outcomes to occur?	Adequacy of follow up of cohorts	
Cenksoy PO 2014	*	*	*	-	**	*	-	*	7
Oral S 2021	*	*	*	-	**	*	*	*	8
Jin BL 2018	*	*	*	-	**	*	*	*	8
Zhang XY 2021	*	*	*	-	**	*	*	*	8
Guo Y 2021	*	*	*	-	-	*	*	*	6
Tu XY 2022	*	*	*	*	*	*	*	*	8
Li Y 2015	*	*	*	-	**	*	*	*	8
Du MZ 2021	*	*	*	-	*	*	*	*	7
Li F 2020	*	*	*	-	**	*	*	*	7
Huang PX 2019	*	*	*	-	**	*	*	*	8
Hu LL 2014	*	*	*	-	*	*	*	*	7
Zhao SB 2023	*	*	*	*	**	*	-	*	7
Li Z 2022	*	*	*	-	**	*	*	*	8
Li WS 2021	*	*	*	*	**	*	*	*	9
Wang MH 2024	*	*	*	*	**	*	*	*	9
A. Racca 2014	*	*	*	*	*	*	*	*	8
Atri Pal 2023	*	*	*	*	-	*	*	*	7
Tarek K 2023	*	*	*	*	-	*	*	*	7
Tzu-Ching Kao 2023	*	*	*	*	*	*	-	*	7

Table S4. PRISMA 2020 checklist.

Section and Topic	Item #	Checklist item	Location where item is reported
**TITLE**			
Title	1	Identify the report as a systematic review.	1
**ABSTRACT**			
Abstract	2	See the PRISMA 2020 for Abstracts checklist.	3
**INTRODUCTION**			
Rationale	3	Describe the rationale for the review in the context of existing knowledge.	5
Objectives	4	Provide an explicit statement of the objective(s) or question(s) the review addresses.	5
**METHODS**			
Eligibility criteria	5	Specify the inclusion and exclusion criteria for the review and how studies were grouped for the syntheses.	7
Information sources	6	Specify all databases, registers, websites, organisations, reference lists and other sources searched or consulted to identify studies. Specify the date when each source was last searched or consulted.	6
Search strategy	7	Present the full search strategies for all databases, registers and websites, including any filters and limits used.	6, Table S1
Selection process	8	Specify the methods used to decide whether a study met the inclusion criteria of the review, including how many reviewers screened each record and each report retrieved, whether they worked independently, and if applicable, details of automation tools used in the process.	9
Data collection process	9	Specify the methods used to collect data from reports, including how many reviewers collected data from each report, whether they worked independently, any processes for obtaining or confirming data from study investigators, and if applicable, details of automation tools used in the process.	7
Data items	10a	List and define all outcomes for which data were sought. Specify whether all results that were compatible with each outcome domain in each study were sought (e.g. for all measures, time points, analyses), and if not, the methods used to decide which results to collect.	9
	10b	List and define all other variables for which data were sought (e.g. participant and intervention characteristics, funding sources). Describe any assumptions made about any missing or unclear information.	7, Table S2
Study risk of bias assessment	11	Specify the methods used to assess risk of bias in the included studies, including details of the tool(s) used, how many reviewers assessed each study and whether they worked independently, and if applicable, details of automation tools used in the process.	8
Effect measures	12	Specify for each outcome the effect measure(s) (e.g. risk ratio, mean difference) used in the synthesis or presentation of results.	8
Synthesis methods	13a	Describe the processes used to decide which studies were eligible for each synthesis (e.g. tabulating the study intervention characteristics and comparing against the planned groups for each synthesis (item #5)).	8
	13b	Describe any methods required to prepare the data for presentation or synthesis, such as handling of missing summary statistics, or data conversions.	8

Section and Topic	Item #	Checklist item	Location where item is reported
	13c	Describe any methods used to tabulate or visually display results of individual studies and syntheses.	8
	13d	Describe any methods used to synthesize results and provide a rationale for the choice(s). If meta-analysis was performed, describe the model(s), method(s) to identify the presence and extent of statistical heterogeneity, and software package(s) used.	8
	13e	Describe any methods used to explore possible causes of heterogeneity among study results (e.g. subgroup analysis, meta-regression).	8
	13f	Describe any sensitivity analyses conducted to assess robustness of the synthesized results.	None
Reporting bias assessment	14	Describe any methods used to assess risk of bias due to missing results in a synthesis (arising from reporting biases).	9
Certainty assessment	15	Describe any methods used to assess certainty (or confidence) in the body of evidence for an outcome.	9
**RESULTS**			
Study selection	16a	Describe the results of the search and selection process, from the number of records identified in the search to the number of studies included in the review, ideally using a flow diagram.	Figure 1
	16b	Cite studies that might appear to meet the inclusion criteria, but which were excluded, and explain why they were excluded.	7-8
Study characteristics	17	Cite each included study and present its characteristics.	Table S2
Risk of bias in studies	18	Present assessments of risk of bias for each included study.	Table S3, Figure S1
Results of individual studies	19	For all outcomes, present, for each study: (a) summary statistics for each group (where appropriate) and (b) an effect estimate and its precision (e.g. confidence/credible interval), ideally using structured tables or plots.	Figure S2
Results of syntheses	20a	For each synthesis, briefly summarise the characteristics and risk of bias among contributing studies.	8-12
	20b	Present results of all statistical syntheses conducted. If meta-analysis was done, present for each the summary estimate and its precision (e.g. confidence/credible interval) and measures of statistical heterogeneity. If comparing groups, describe the direction of the effect.	8-12
	20c	Present results of all investigations of possible causes of heterogeneity among study results.	13
	20d	Present results of all sensitivity analyses conducted to assess the robustness of the synthesized results.	None
Reporting biases	21	Present assessments of risk of bias due to missing results (arising from reporting biases) for each synthesis assessed.	14
Certainty of evidence	22	Present assessments of certainty (or confidence) in the body of evidence for each outcome assessed.	9-12
**DISCUSSION**			
Discussion	23a	Provide a general interpretation of the results in the context of other evidence.	15
	23b	Discuss any limitations of the evidence included in the review.	15

Section and Topic	Item #	Checklist item	Location where item is reported
	23c	Discuss any limitations of the review processes used.	15
	23d	Discuss implications of the results for practice, policy, and future research.	15
**OTHER INFORMATION**			
Registration and protocol	24a	Provide registration information for the review, including register name and registration number, or state that the review was not registered.	6
	24b	Indicate where the review protocol can be accessed, or state that a protocol was not prepared.	6
	24c	Describe and explain any amendments to information provided at registration or in the protocol.	6
Support	25	Describe sources of financial or non-financial support for the review, and the role of the funders or sponsors in the review.	1
Competing interests	26	Declare any competing interests of review authors.	1
Availability of data, code and other materials	27	Report which of the following are publicly available and where they can be found: template data collection forms; data extracted from included studies; data used for all analyses; analytic code; any other materials used in the review.	1

Figure S1. Risk of bias across through all studies included RCTs.

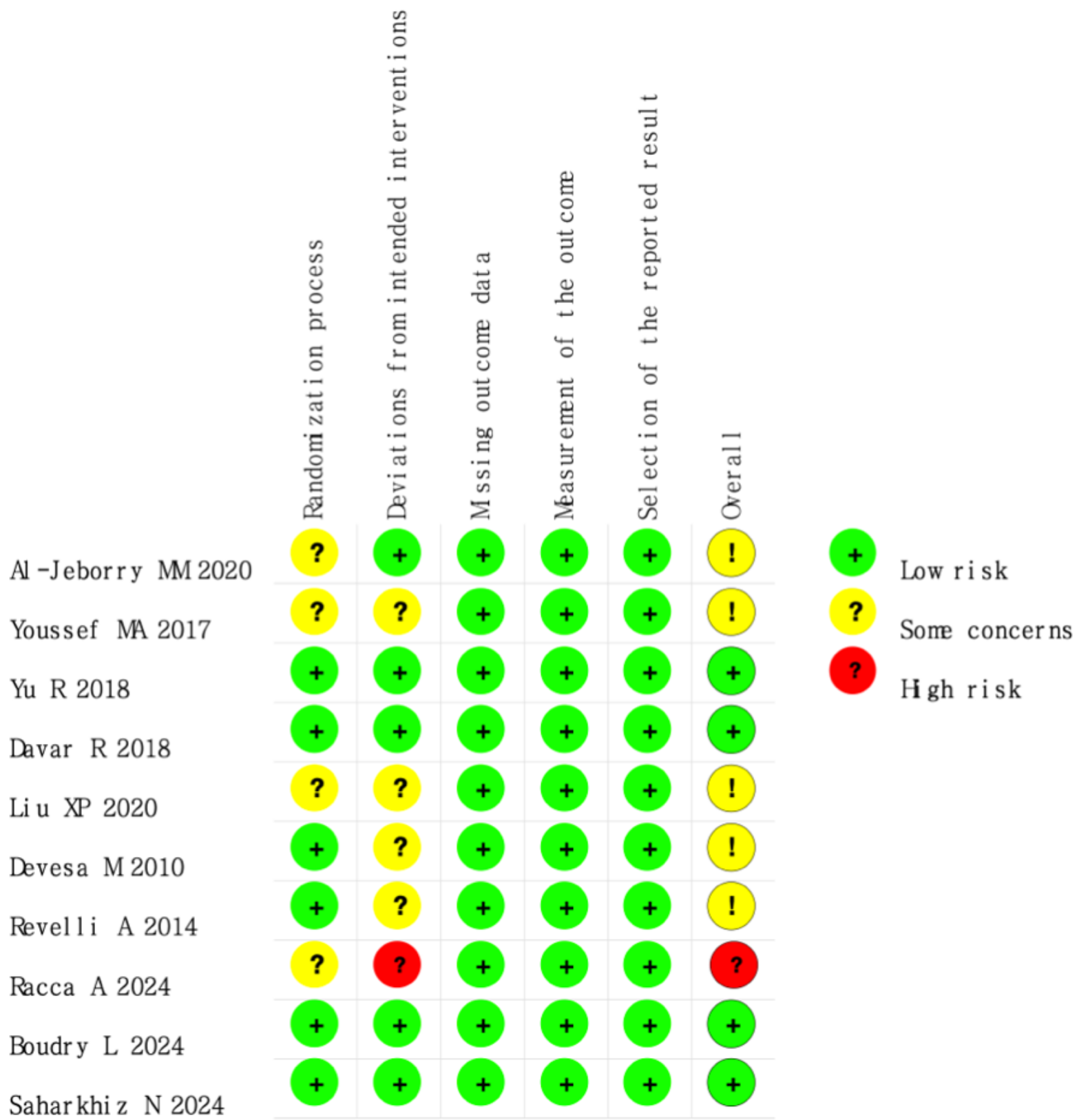

Figure S2. The network plots for the primary and secondary outcomes.

Outcomes	Bologna group	POSEIDON group 3	POSEIDON group 4
**Live birth rate**	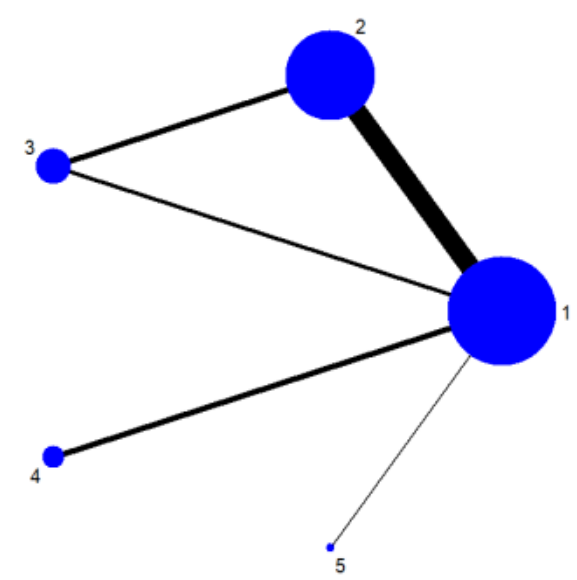	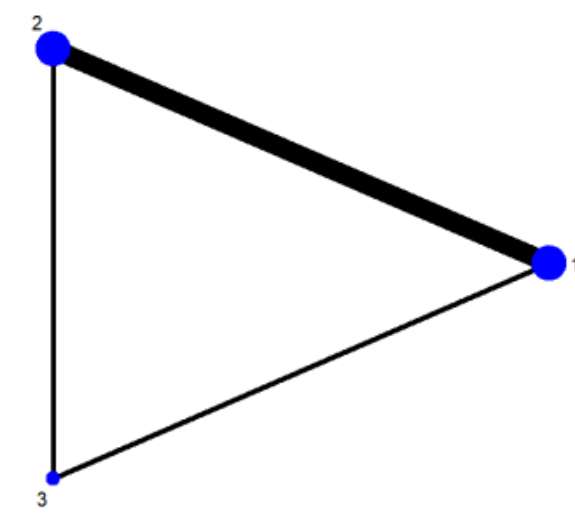	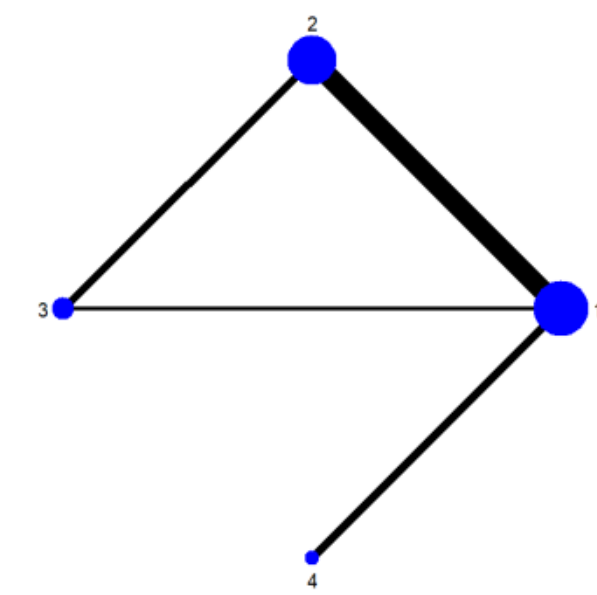
**Cumulative Live birth rate**	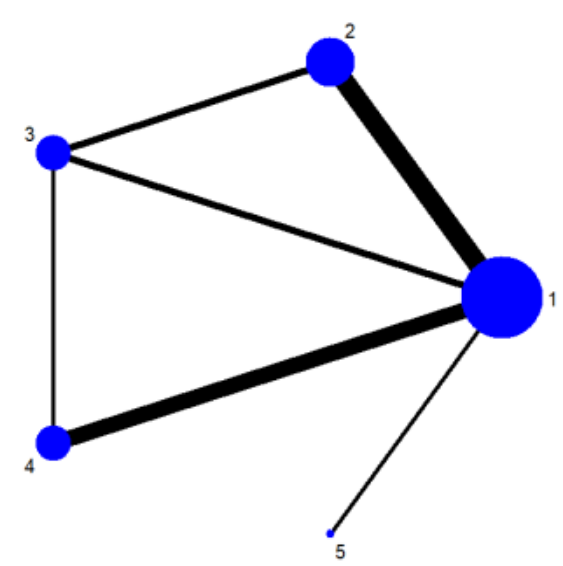	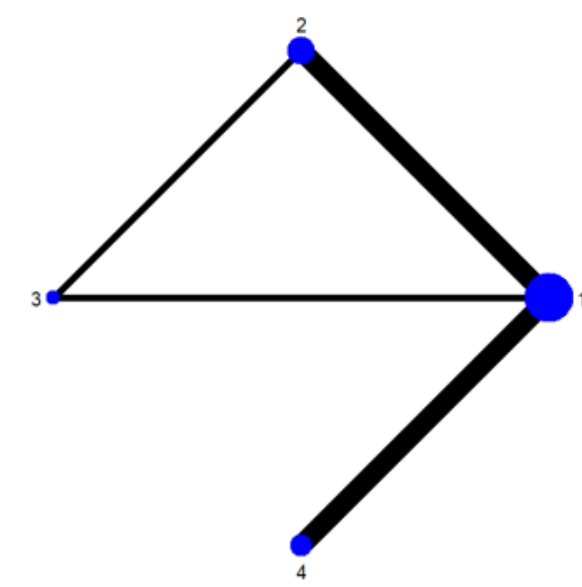	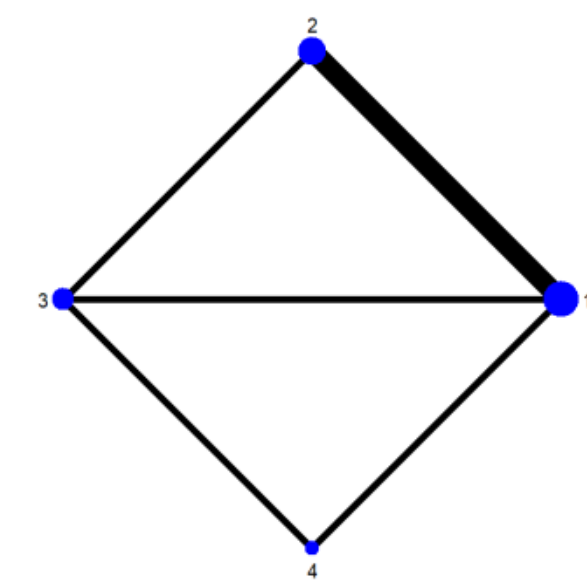
**Clinical pregnancy rate**	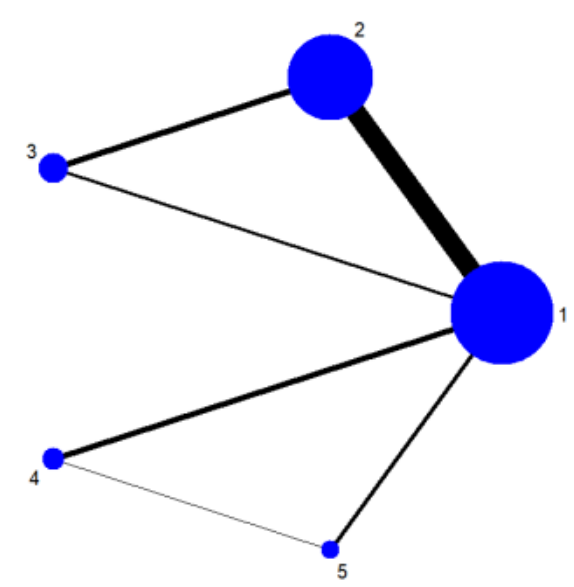	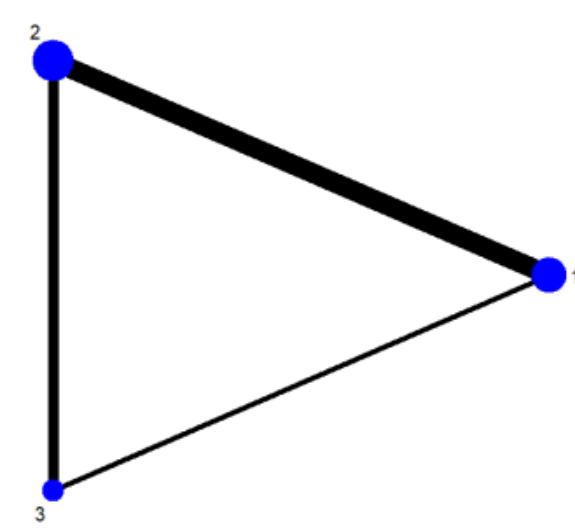	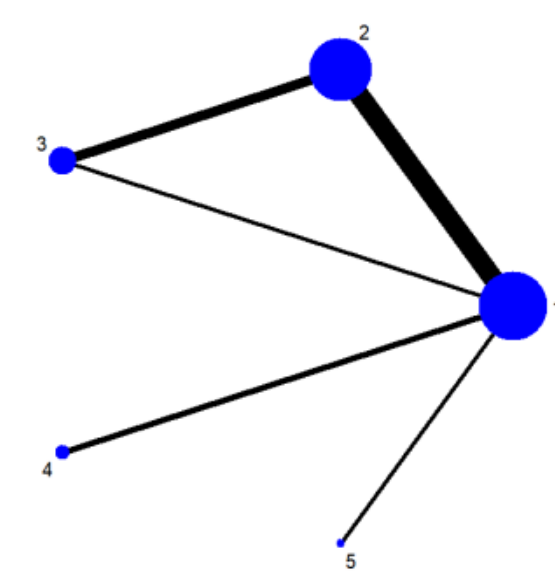

**The number of oocytes retrieved**	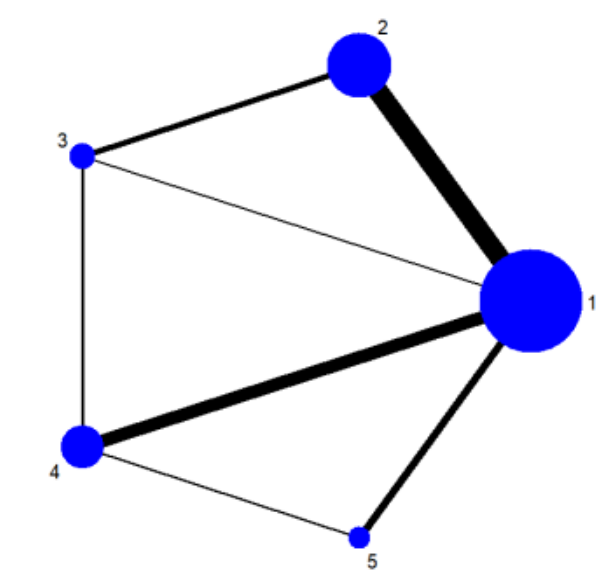	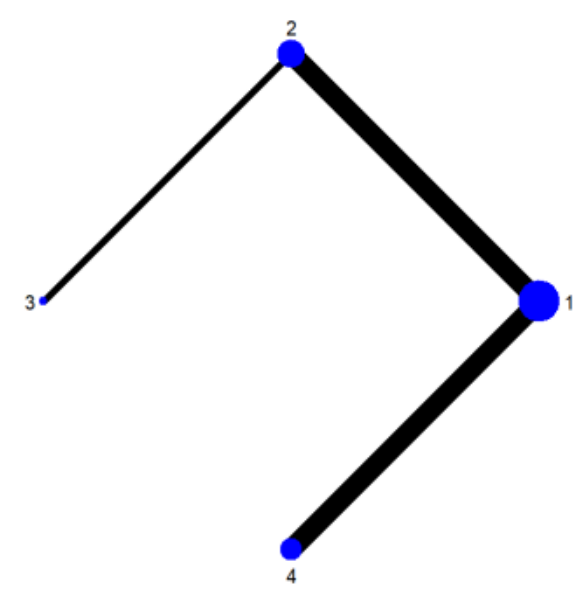	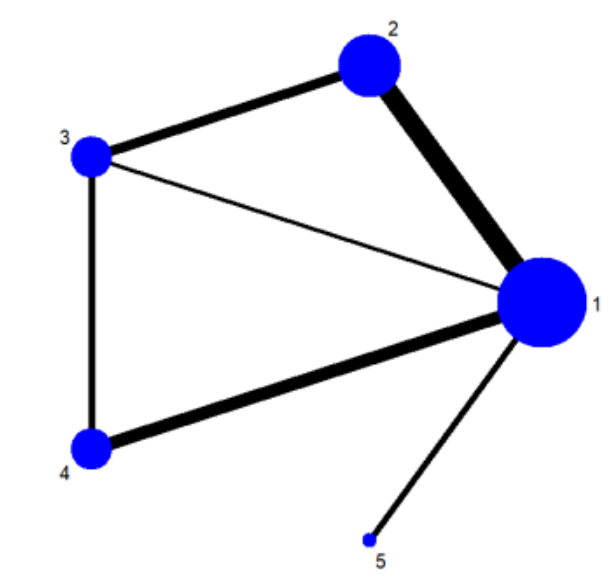
**Cycle cancellation rate**	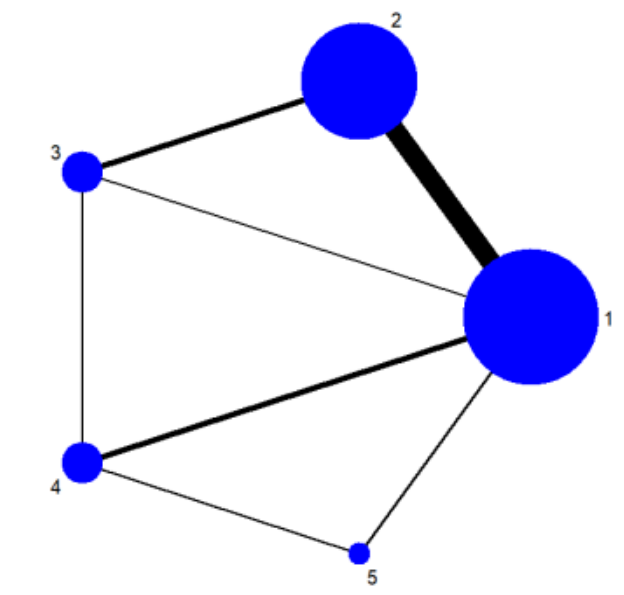	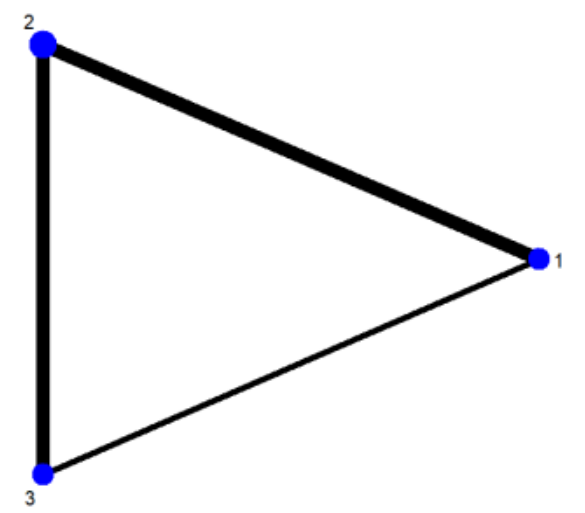	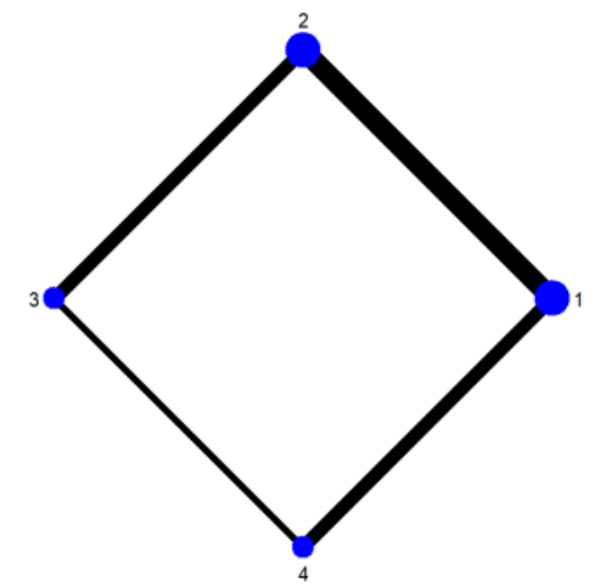

1. GnRH antagonist; 2. GnRH agonist; 3. Mild stimulation; 4. PPOS; 5. Dual ovarian stimulation.

Figure S3. Surface under the cumulative ranking curve for the primary and secondary outcomes.

<table>
<tr><th>Outcomes</th><th>Bologna group</th><th>POSEIDON group 3</th><th>POSEIDON group 4</th></tr>
<tr><td>Live birth rate</td>
<td>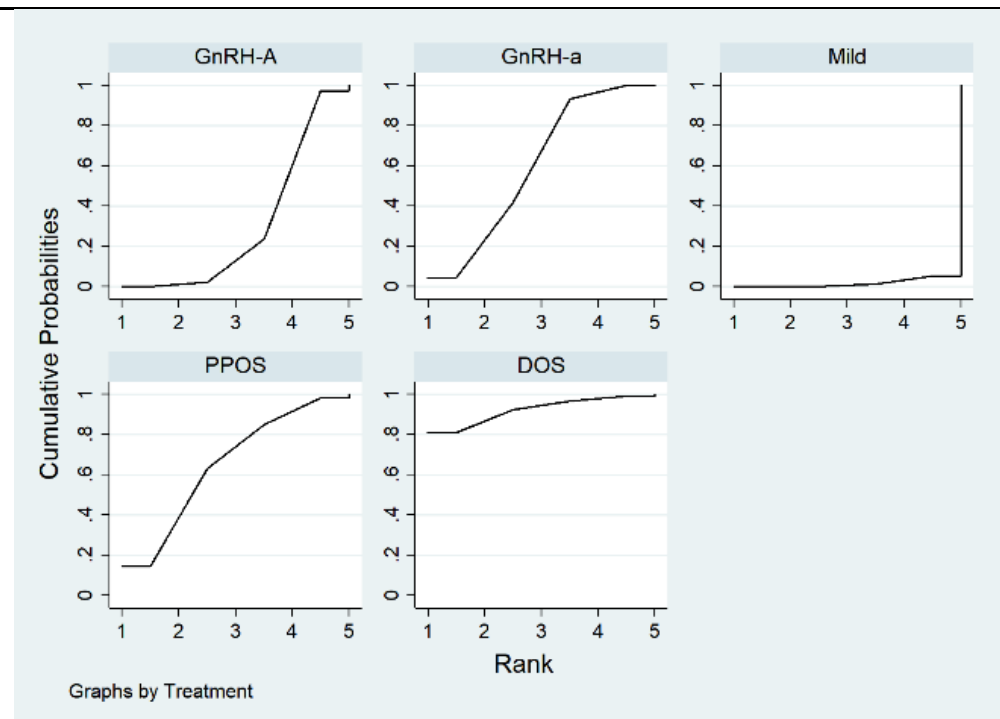
</td>
<td>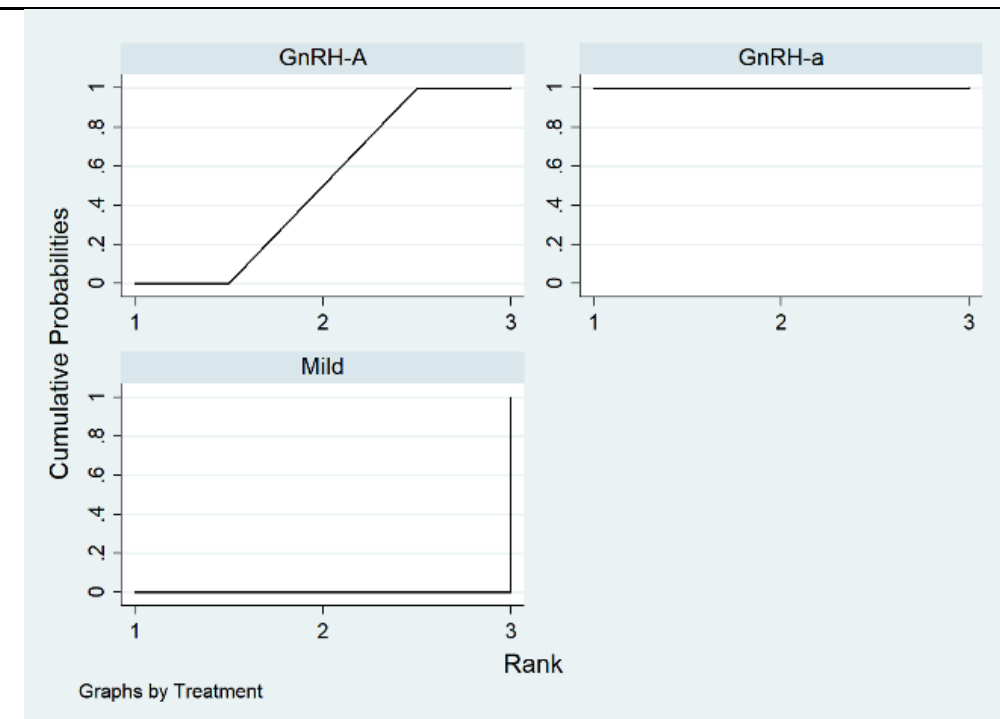
</td>
<td>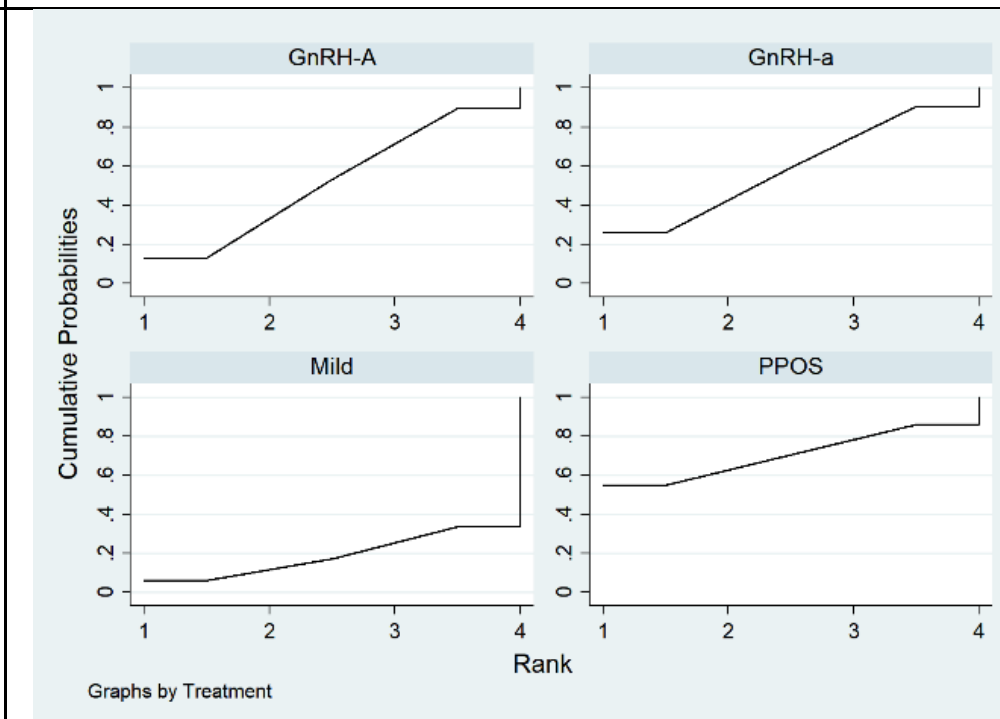
</td></tr>
<tr><td>Cumulative Live birth rate</td>
<td>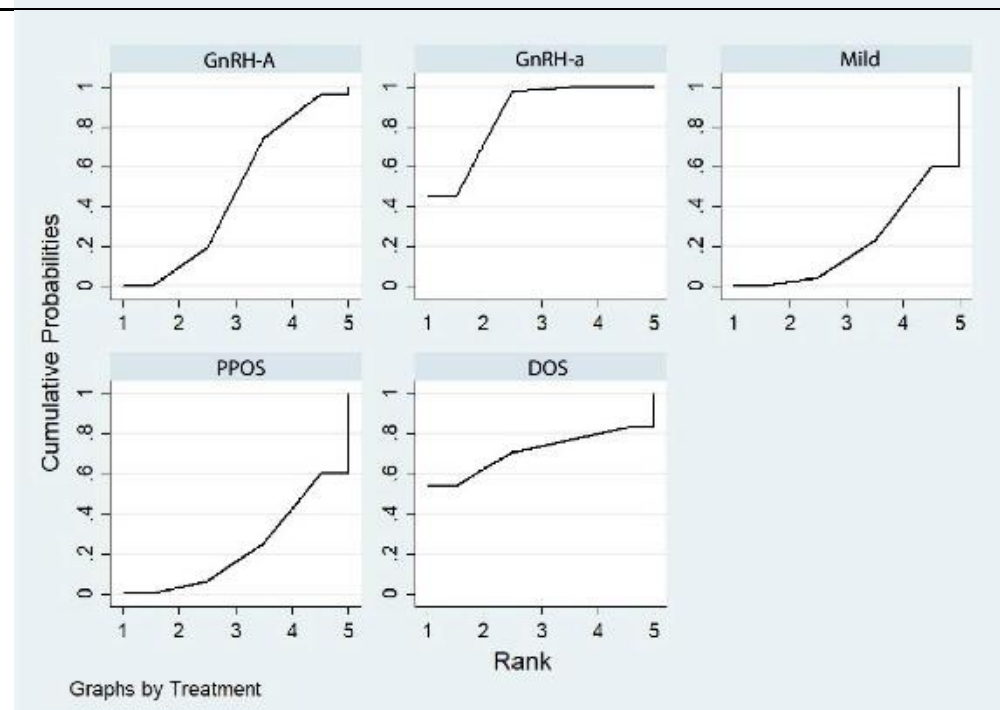
</td>
<td>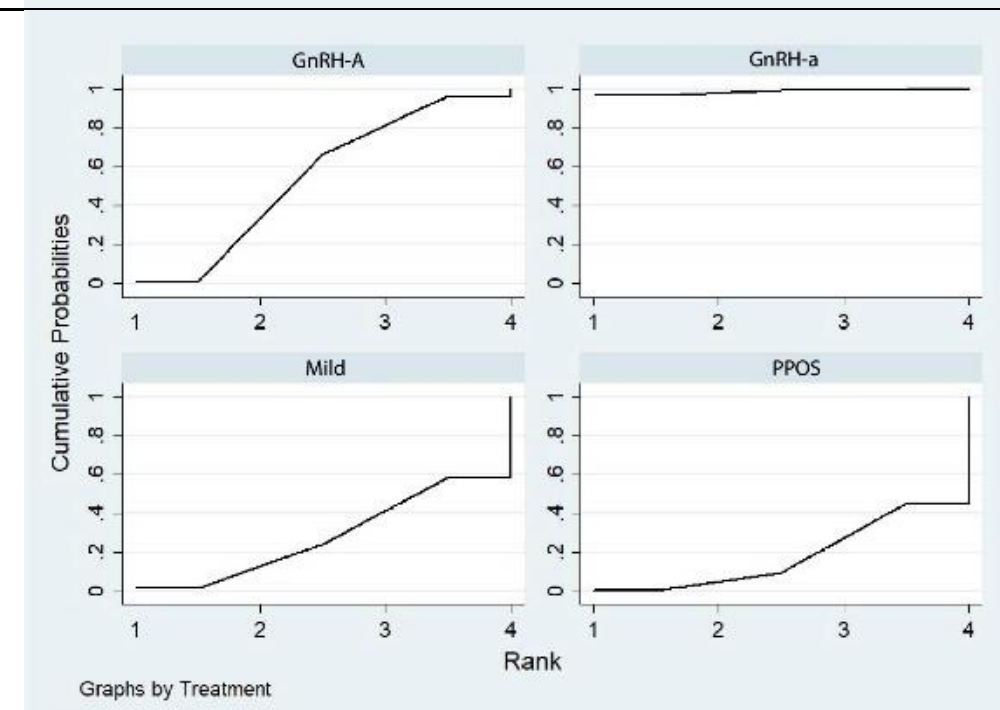
</td>
<td>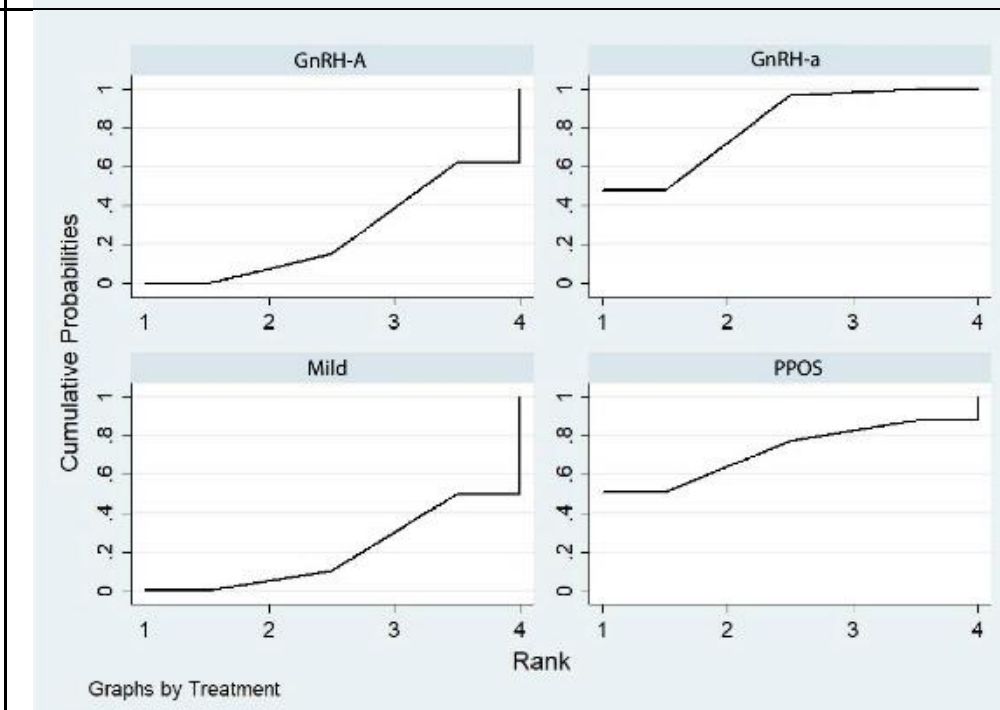
</td></tr>
<tr><td>Clinical pregnancy rate</td>
<td>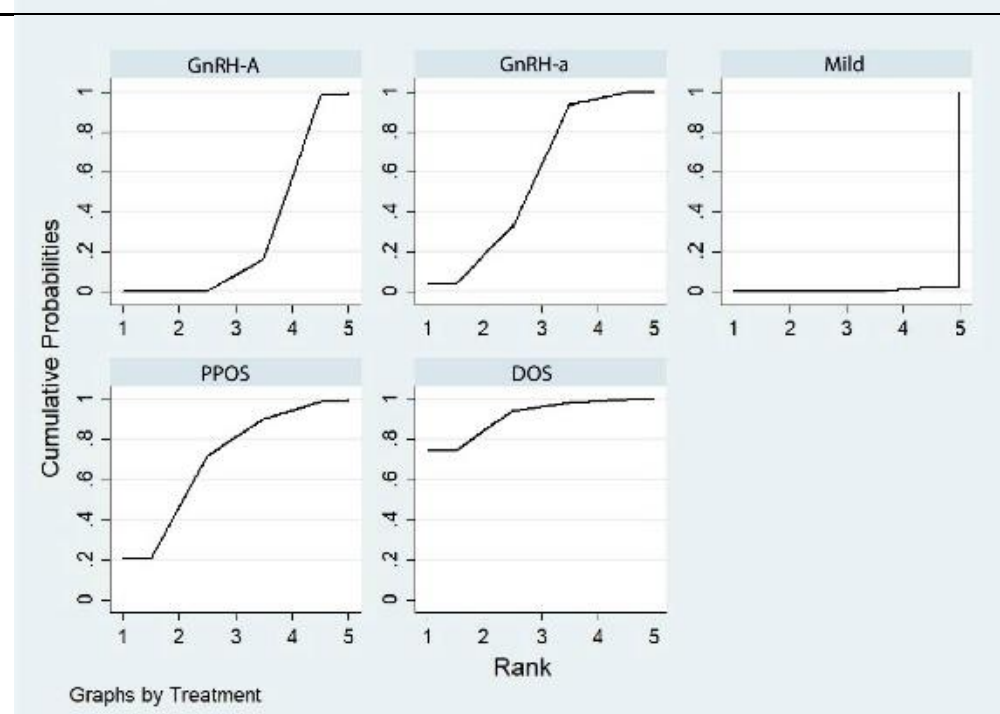
</td>
<td>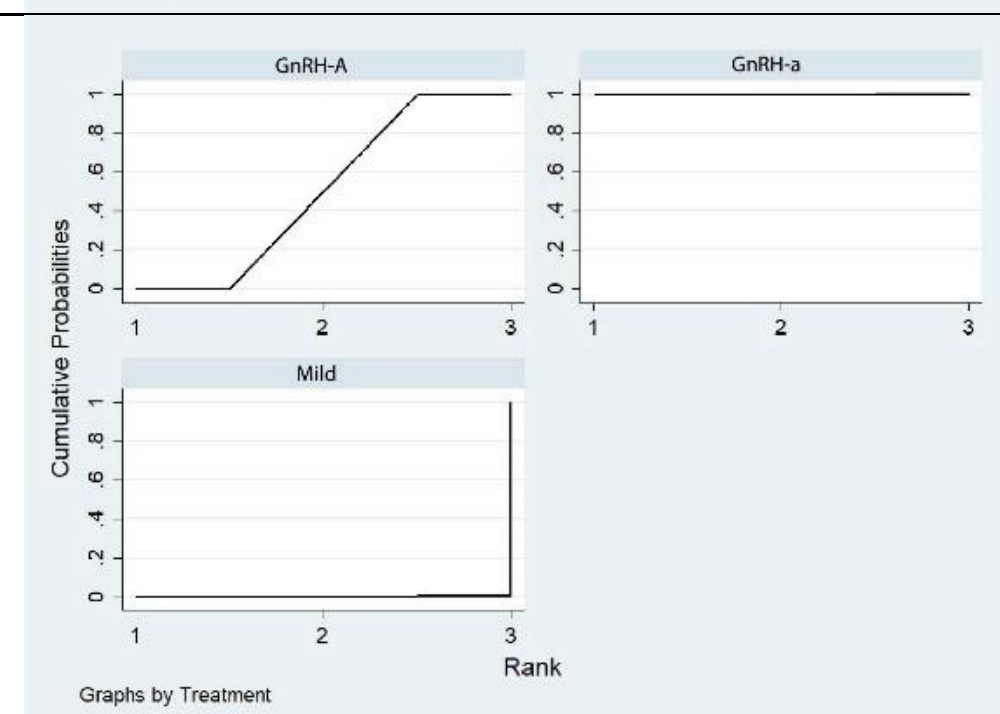
</td>
<td>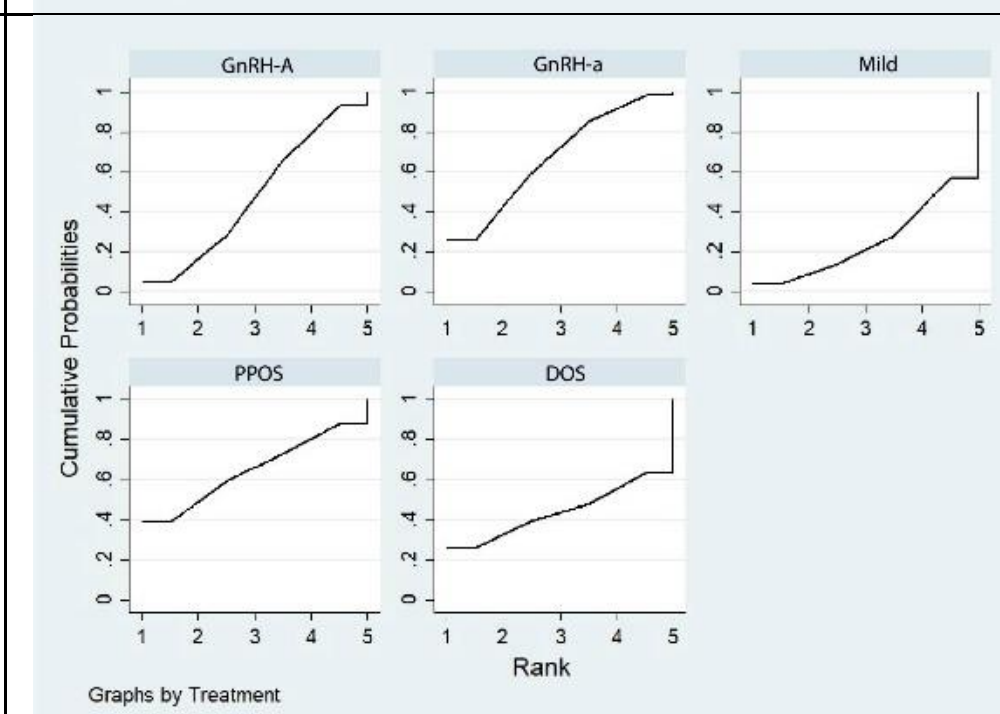
</td></tr>
</table>

<table>
<tr>
<td>The number of oocytes retrieved</td>
<td>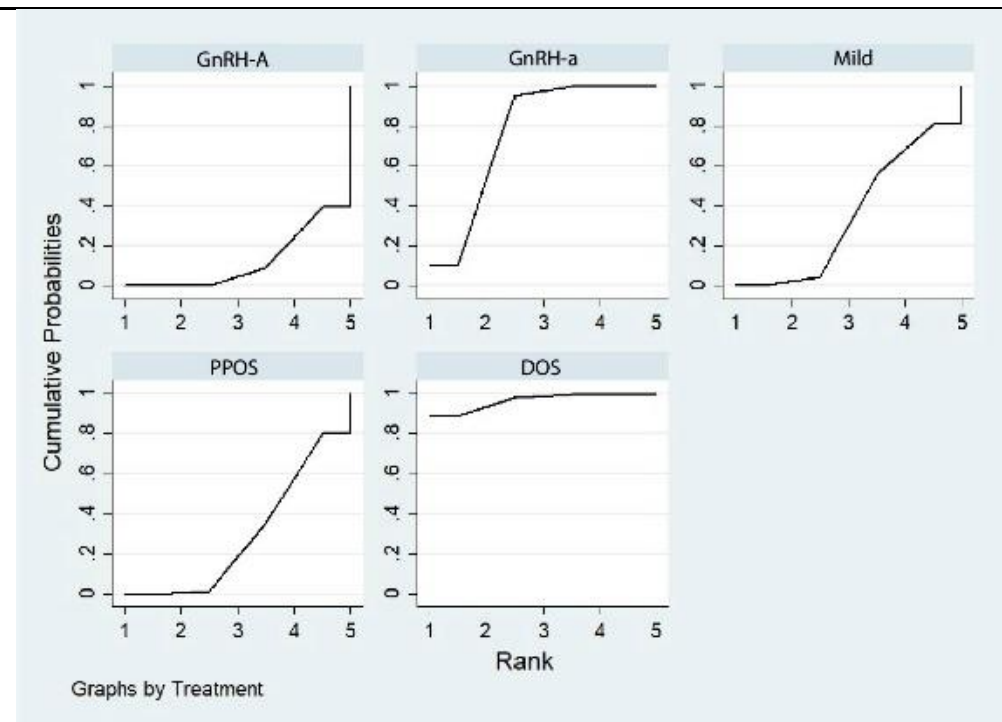
</td>
<td>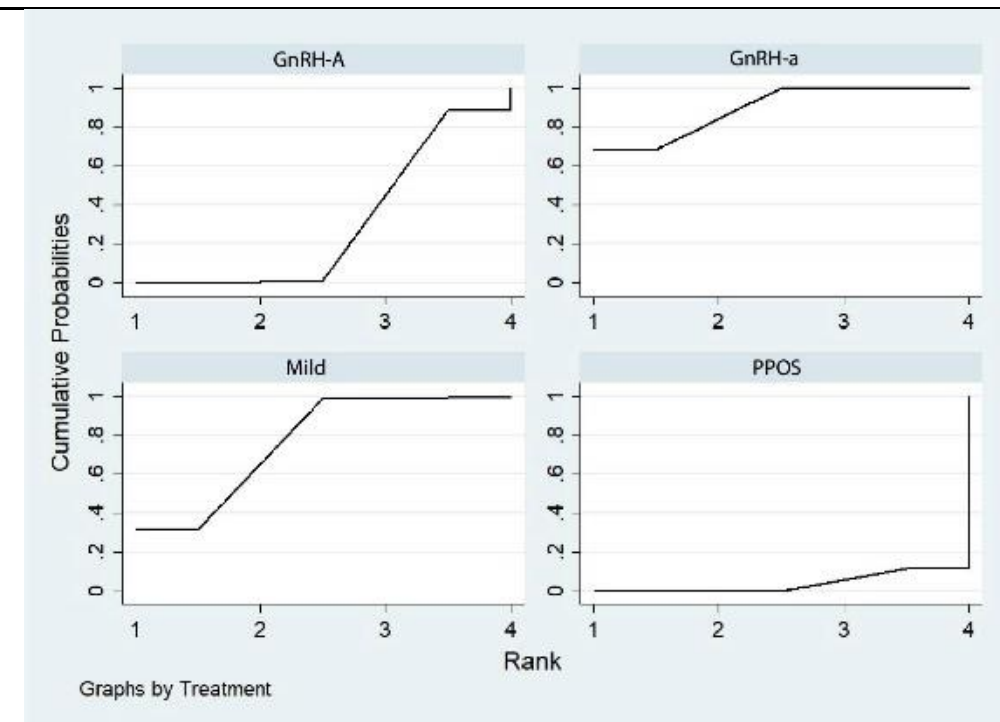
</td>
<td>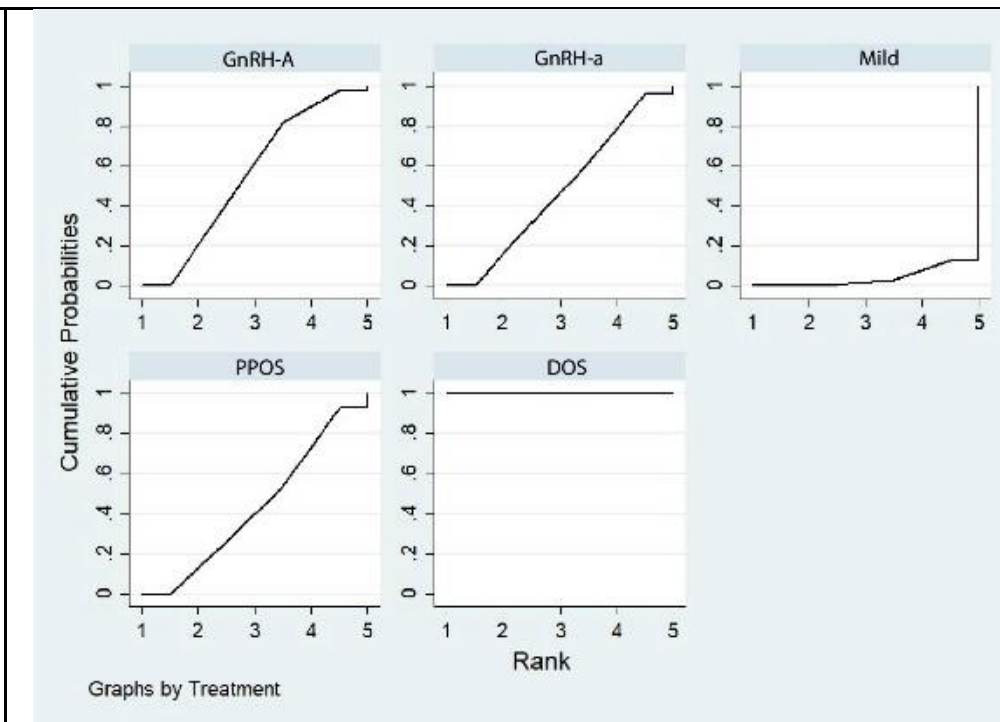
</td>
</tr>
<tr>
<td>Cycle cancellation rate</td>
<td>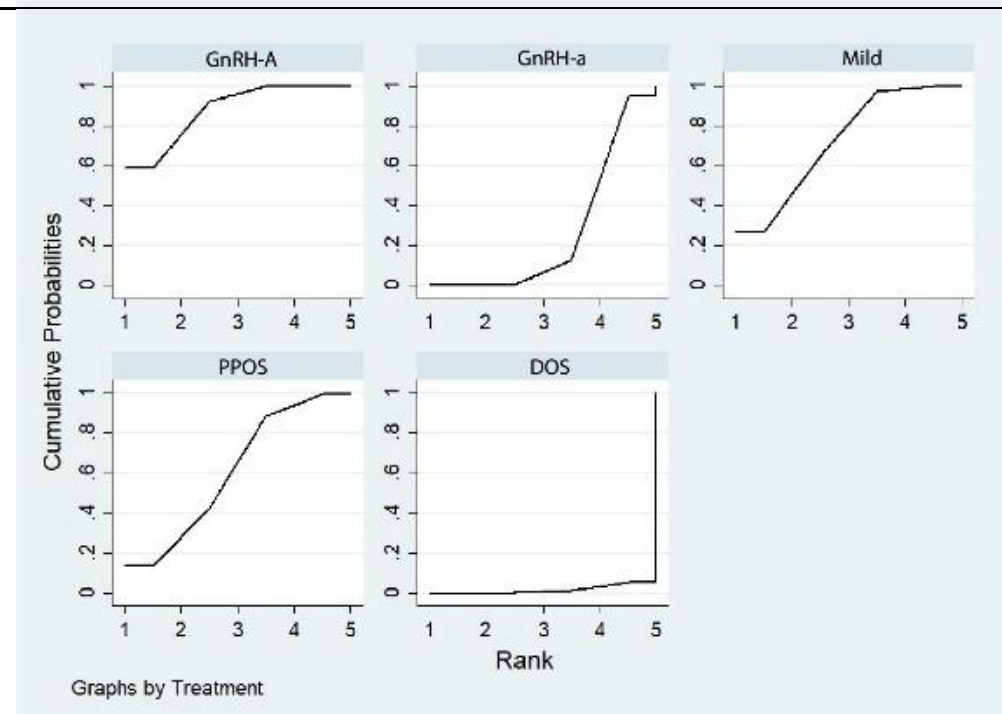
</td>
<td>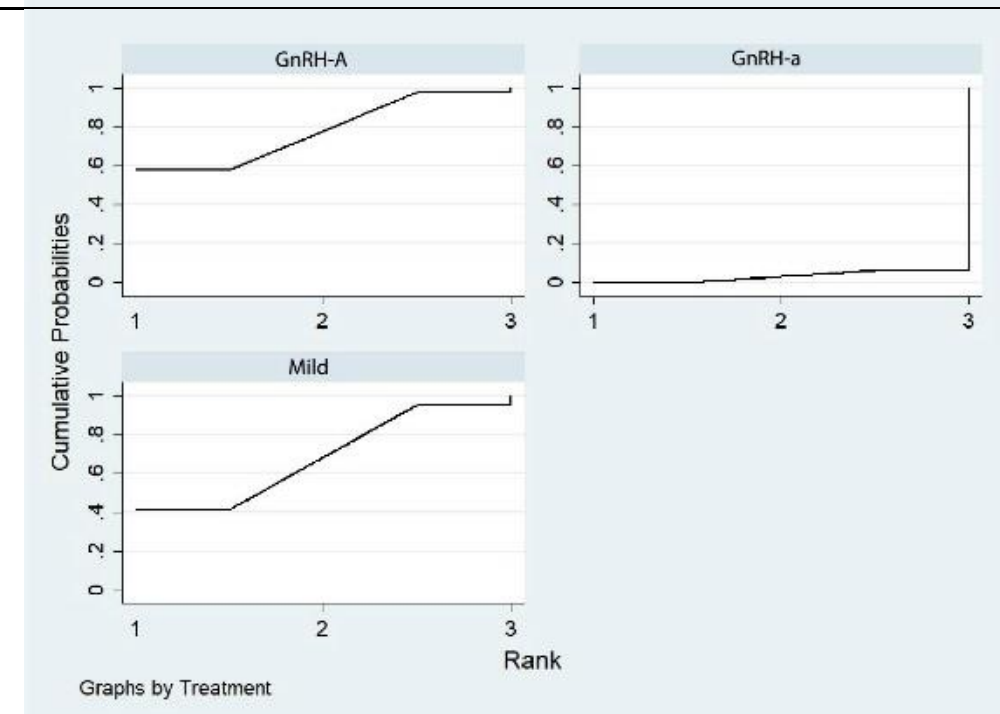
</td>
<td>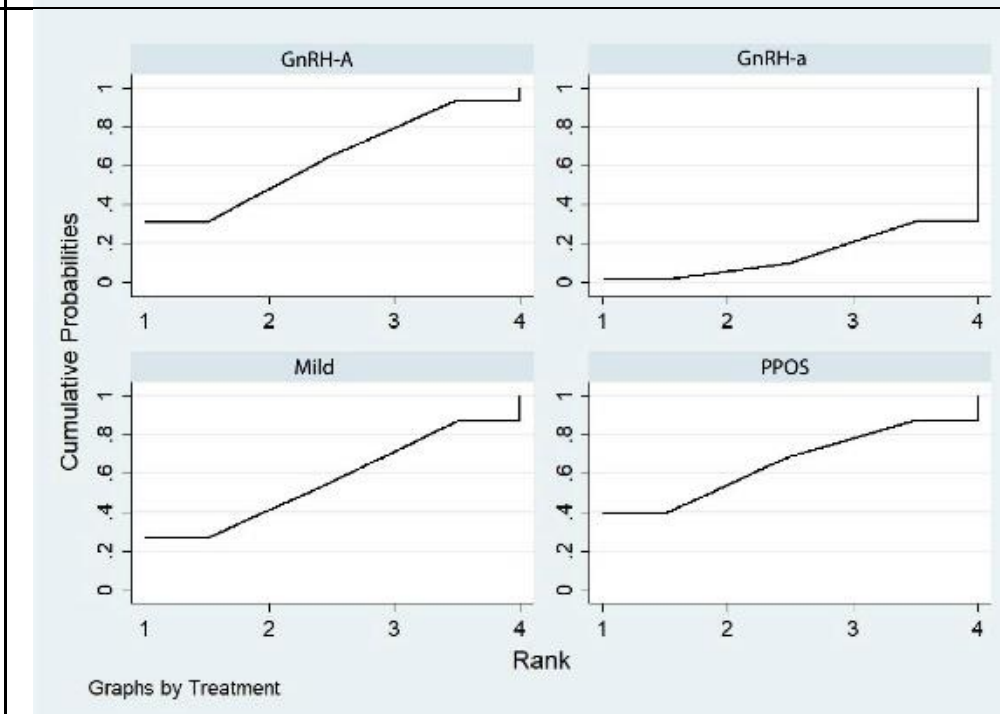
</td>
</tr>
</table>

Figure S4. Comparison-adjusted funnel plot for the primary and secondary outcomes.

Outcomes	Bologna group	POSEIDON group 3	POSEIDON group 4
Live birth rate	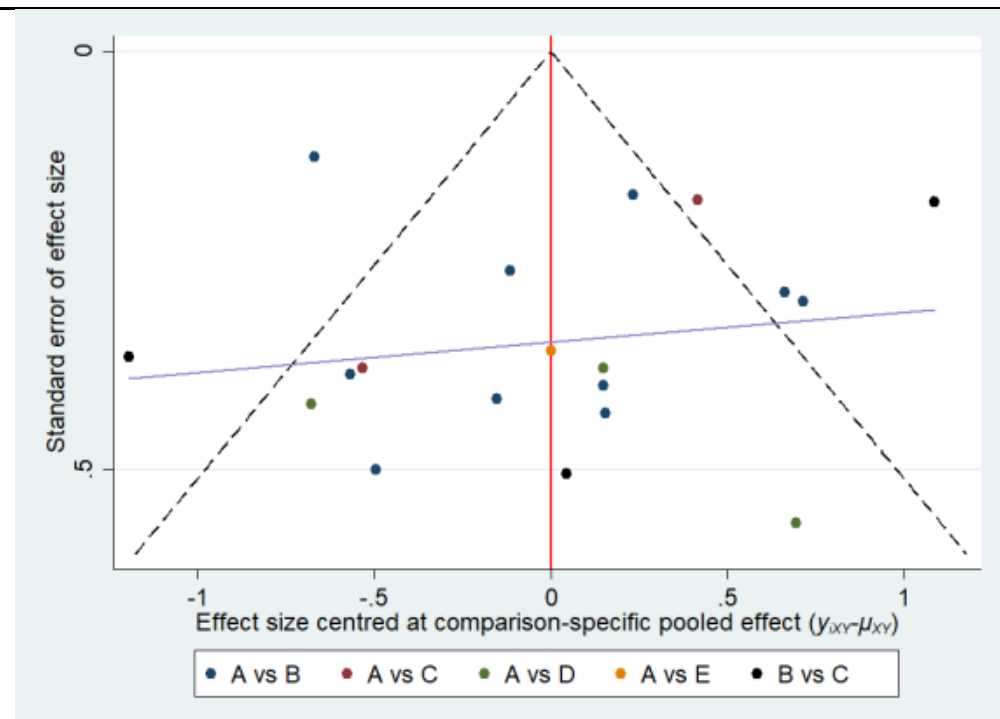	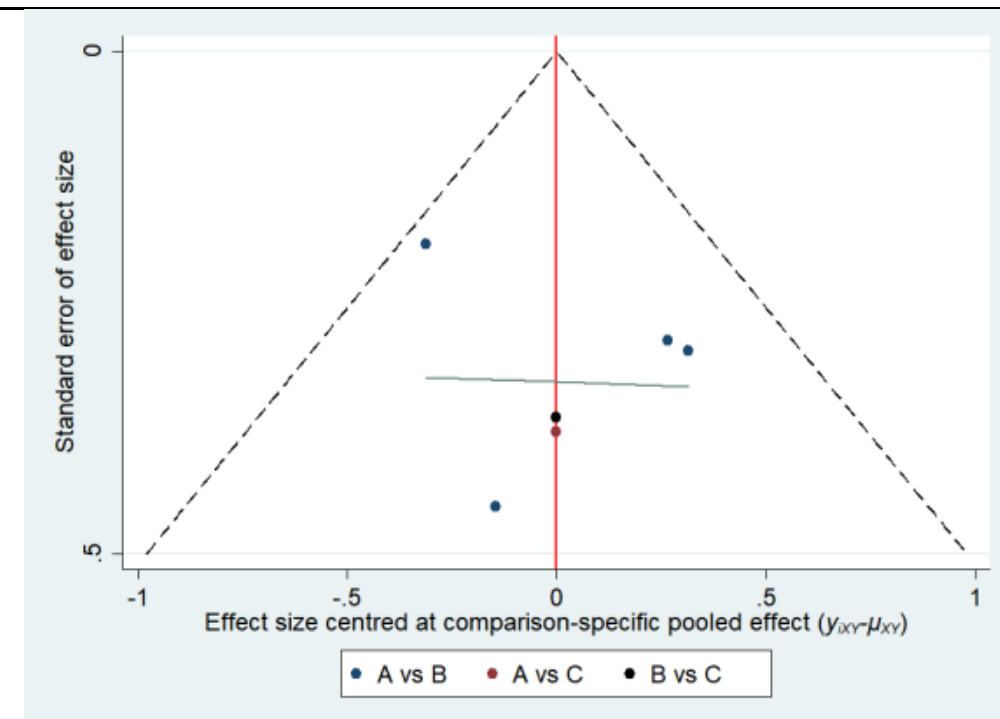	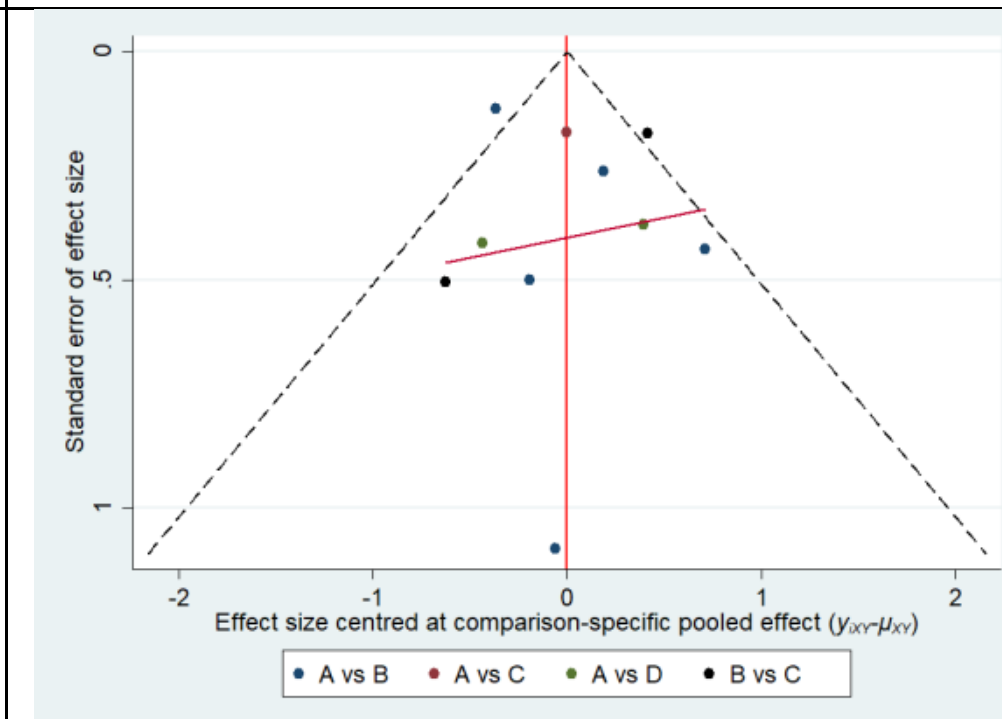
Cumulative Live birth rate	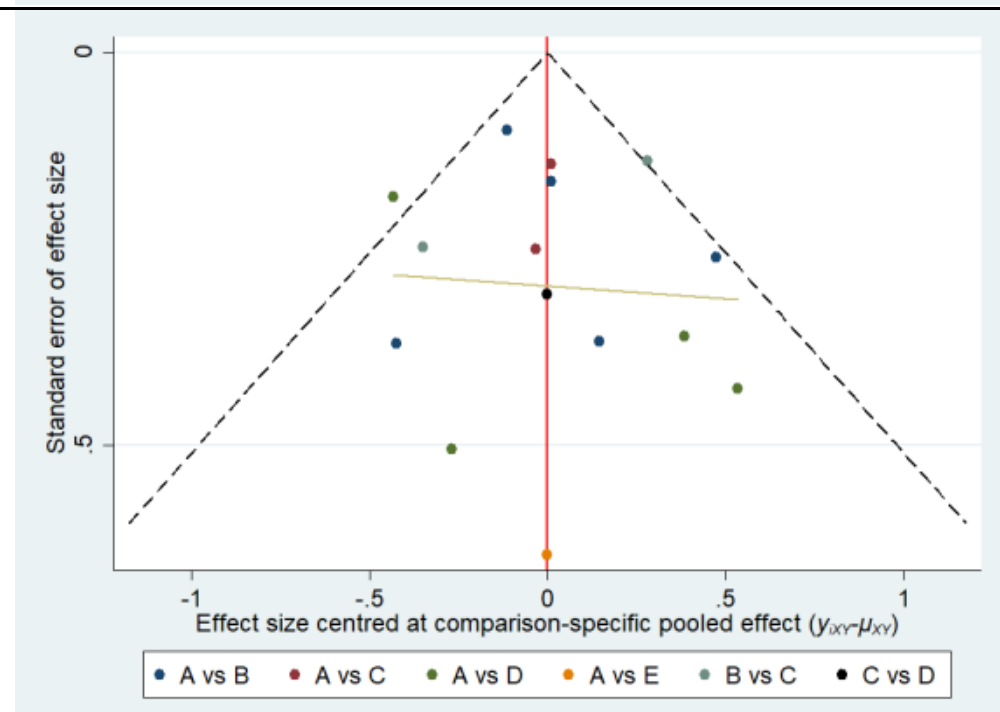	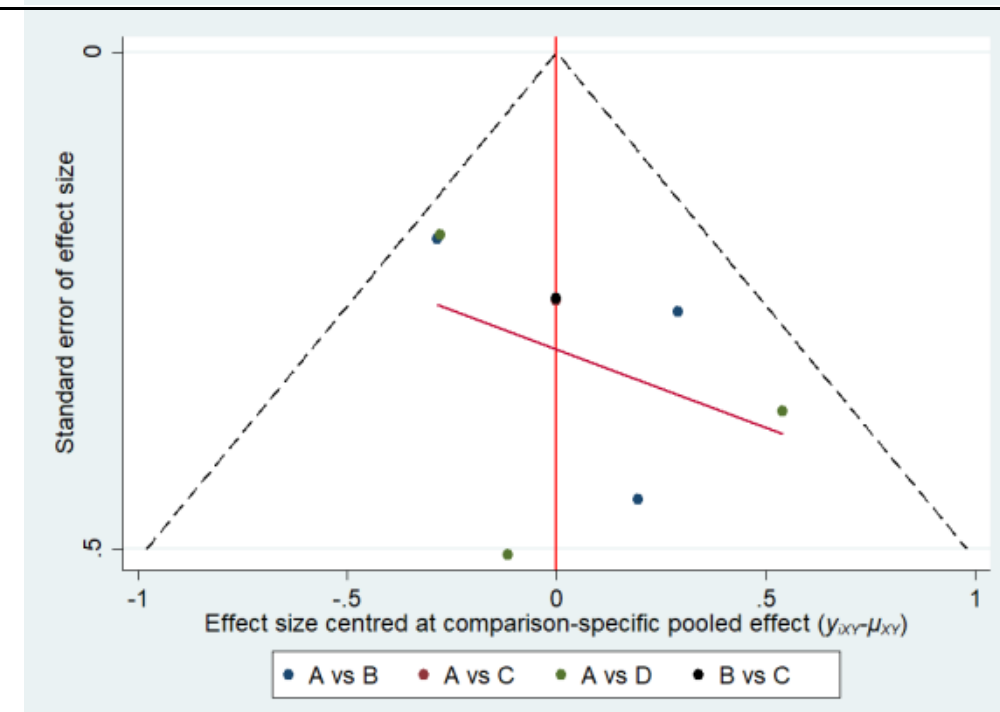	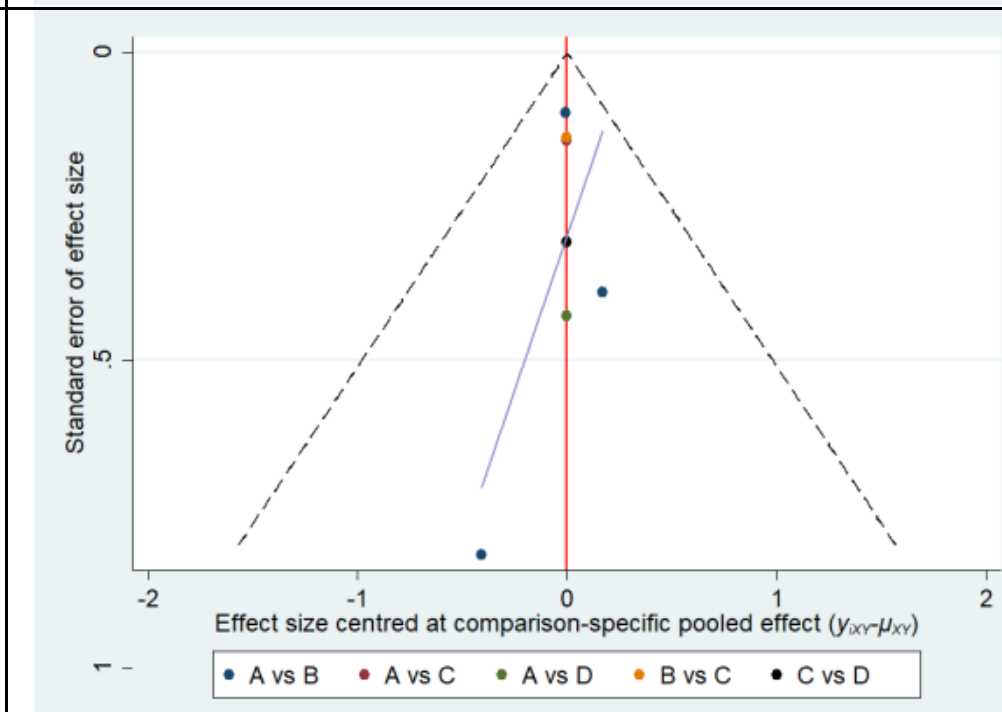
Clinical pregnancy rate	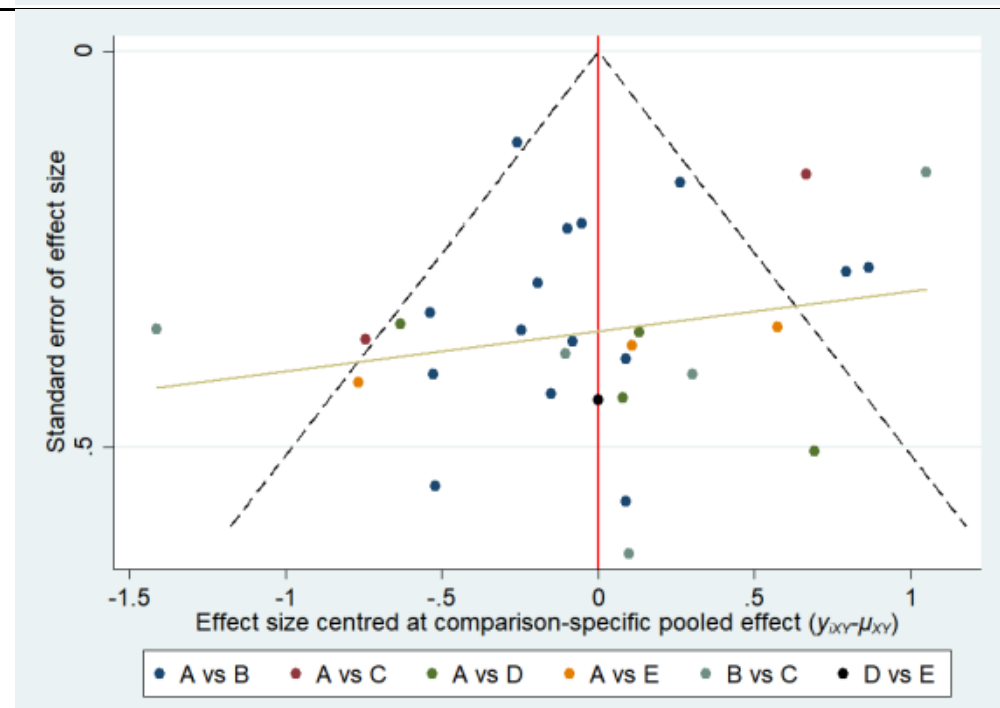	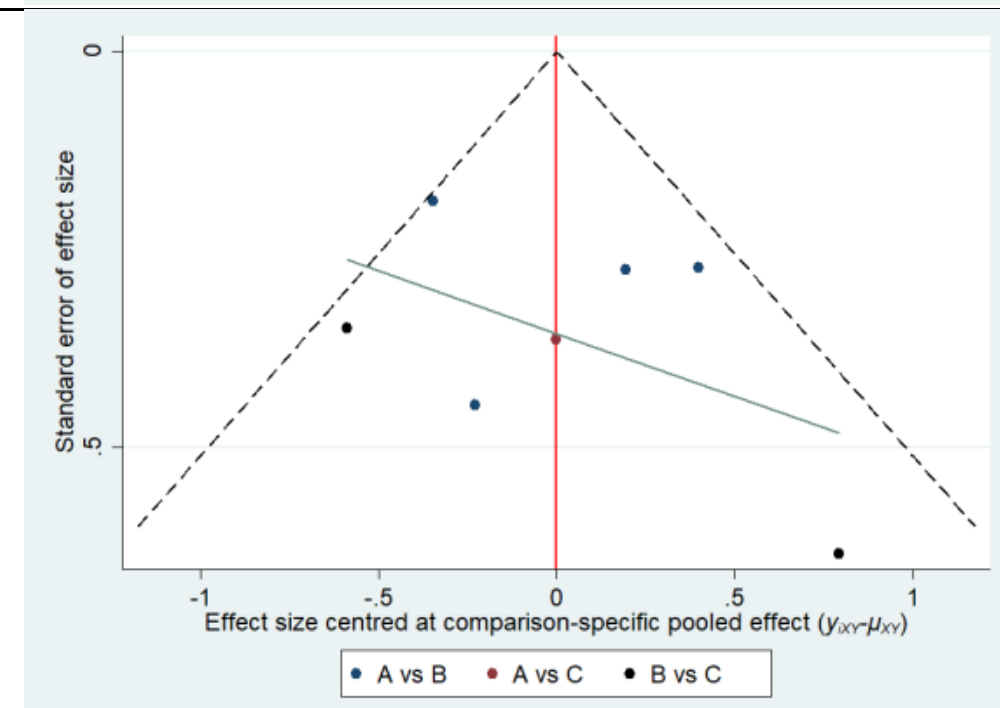	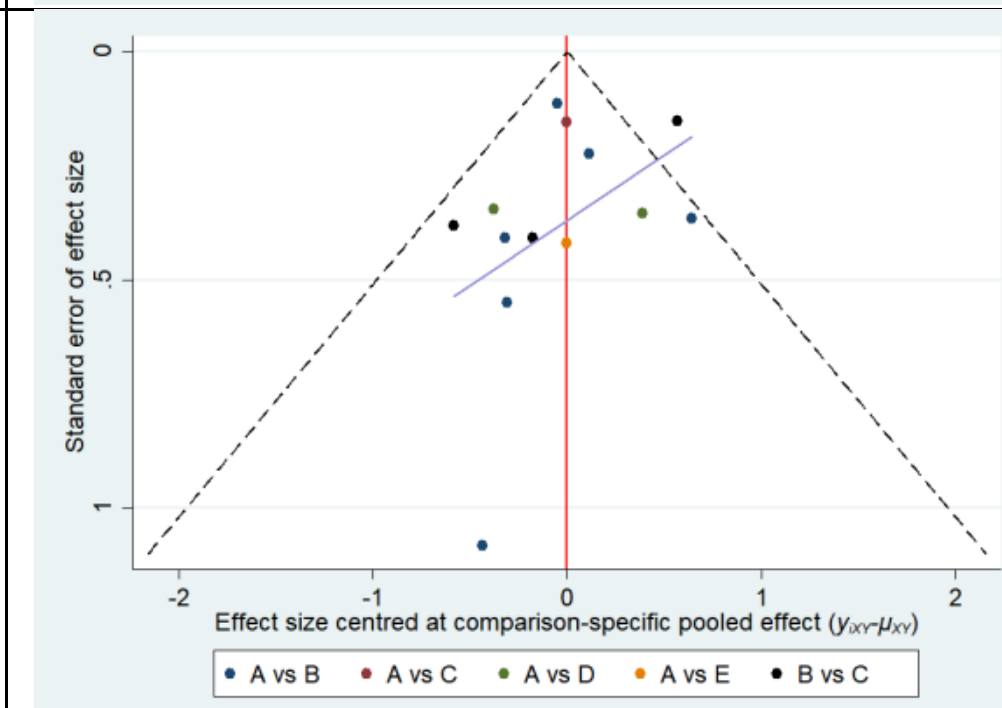

The number of oocytes retrieved

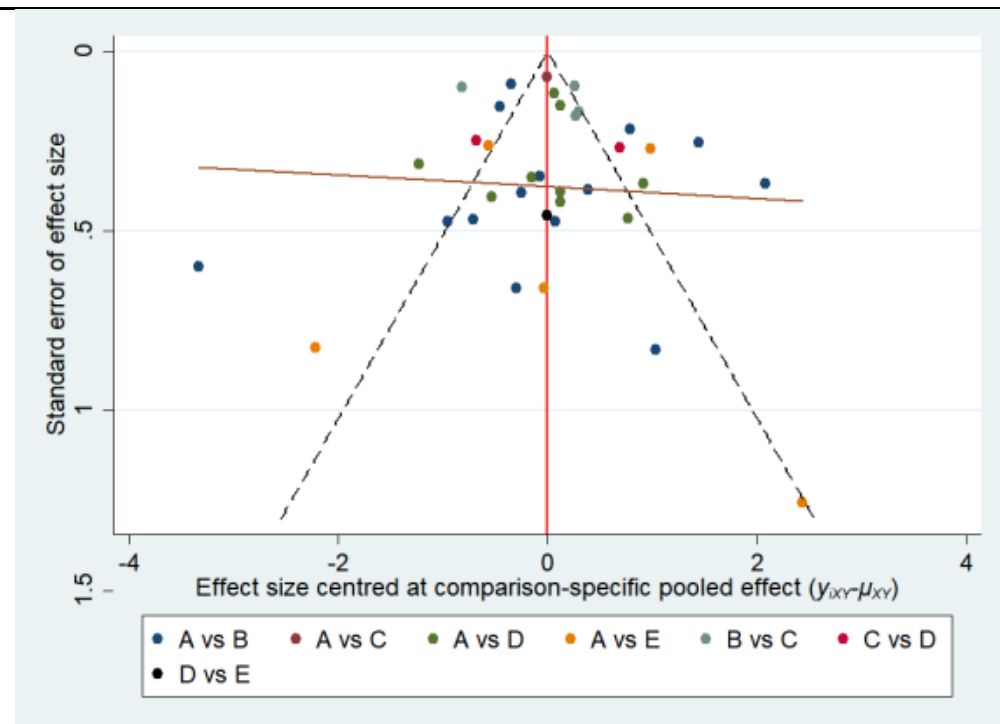

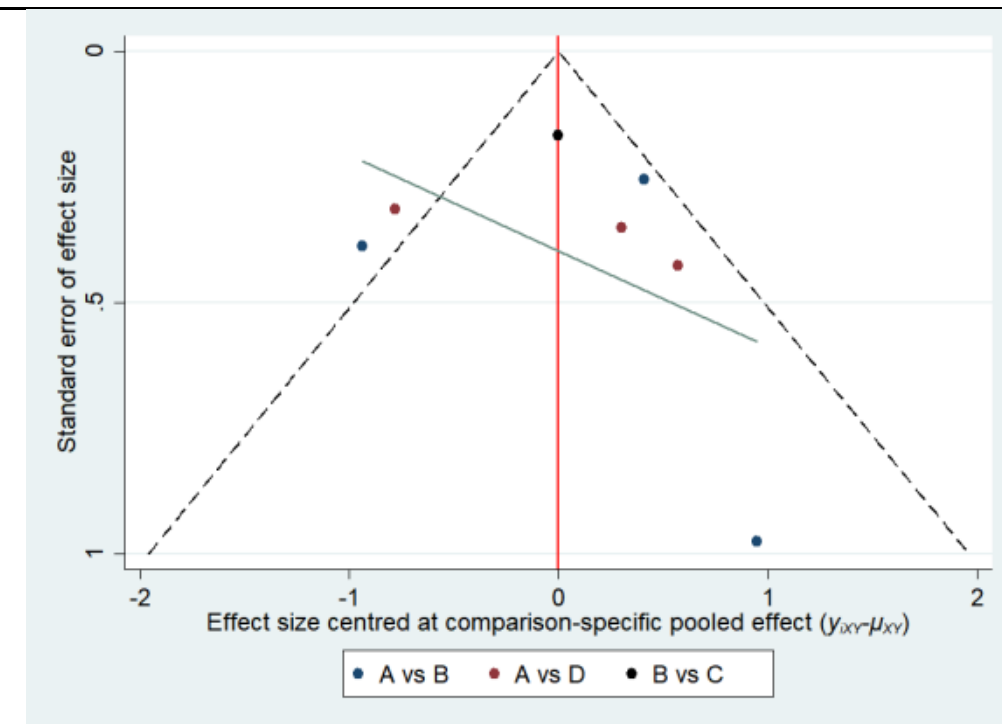

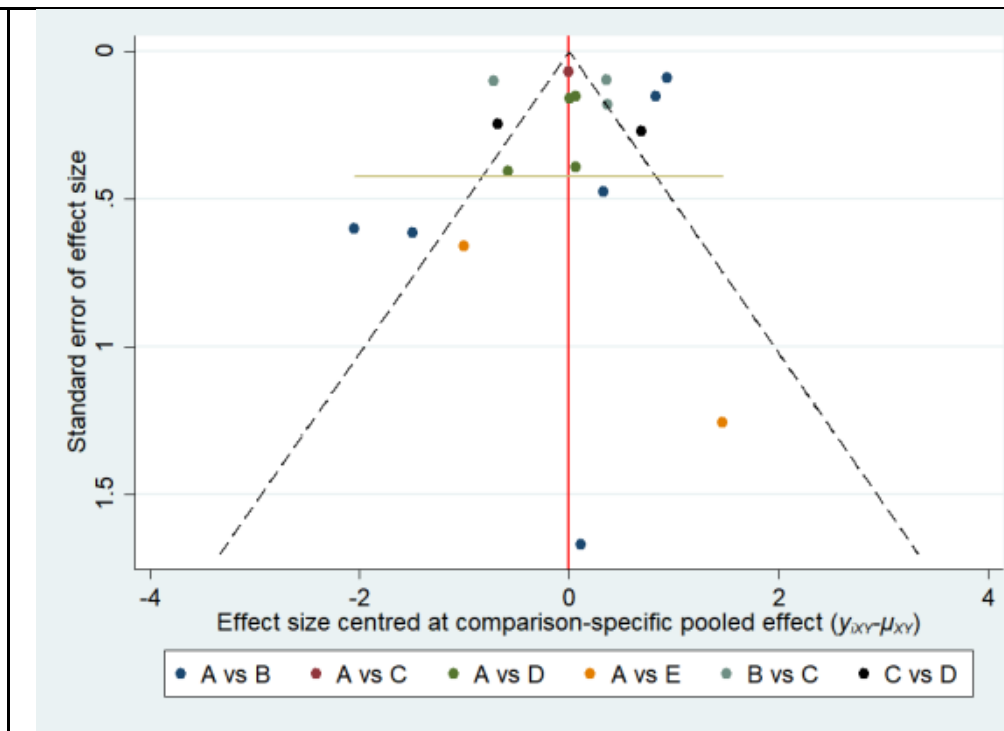

Cycle cancellation rate

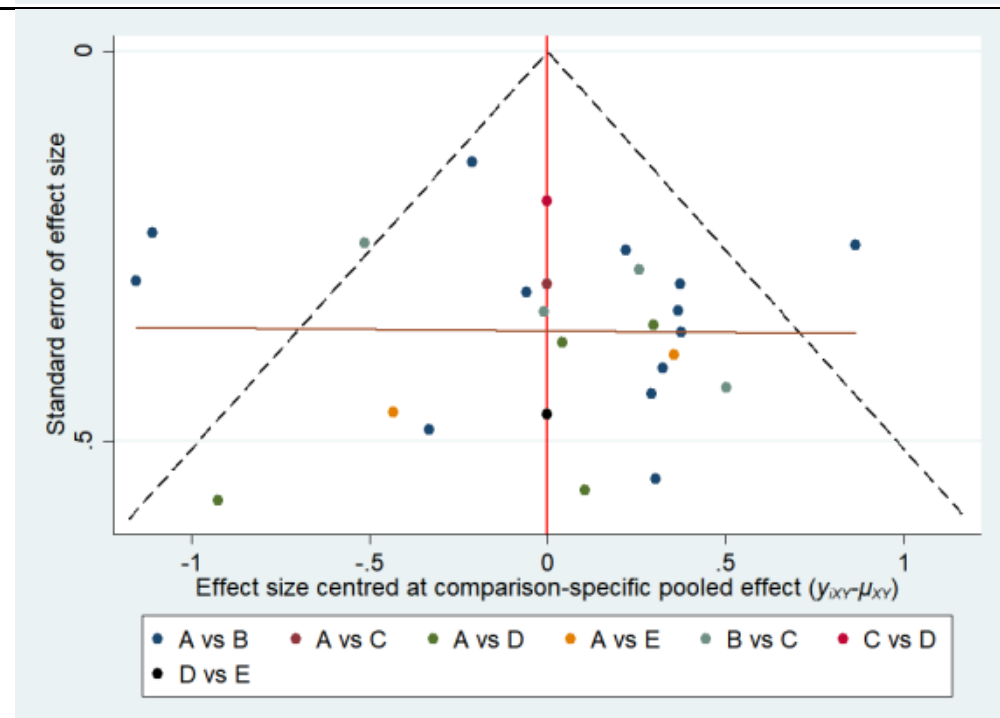

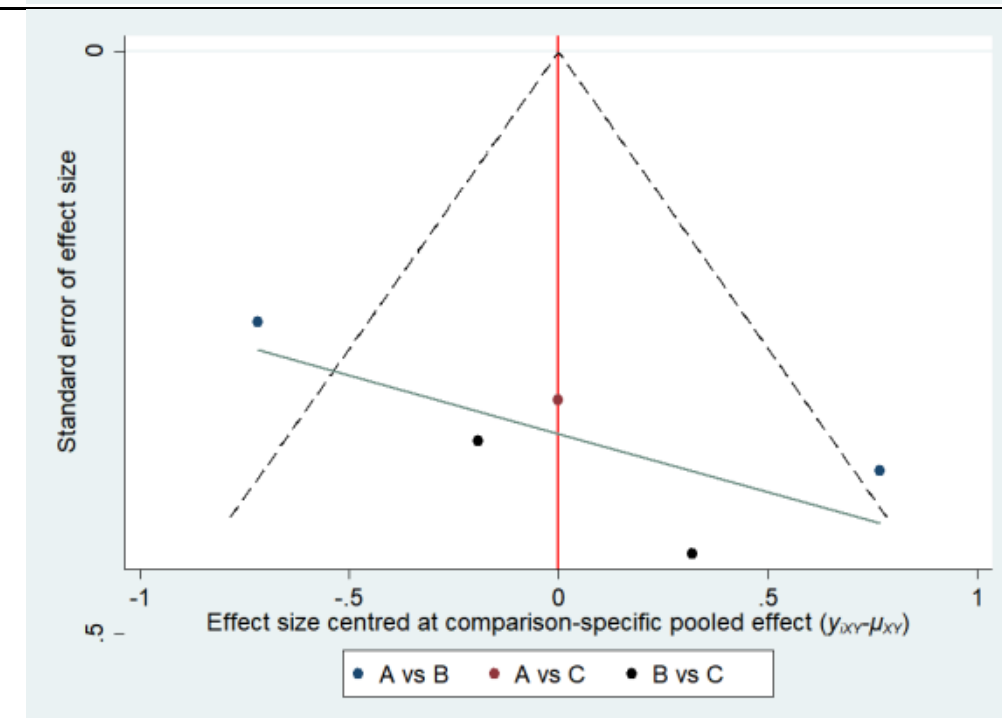

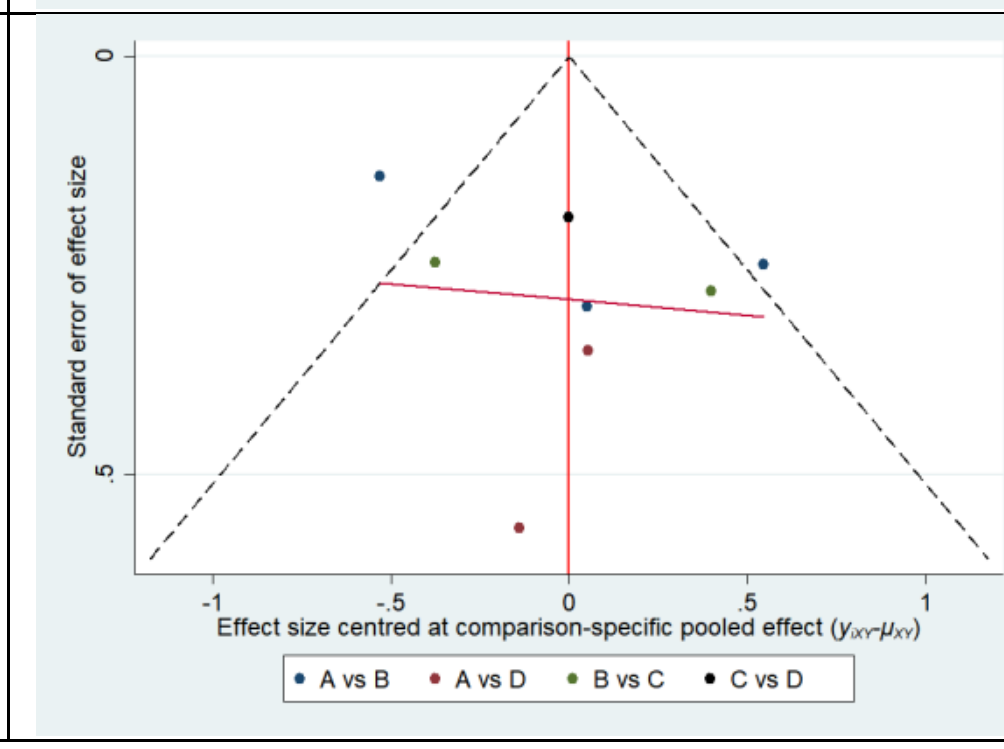

A. GnRH antagonist; B. GnRH agonist; C. Mild stimulation; D. PPOS; E. Dual ovarian stimulation.
